# Febrile Seizure Team-based Learning

**DOI:** 10.21980/J8JD12

**Published:** 2020-10-15

**Authors:** Mary Jane Piroutek

**Affiliations:** * Children’s Hospital of Orange County, Department of Emergency Medicine, Orange, CA

## Abstract

**Audience:**

This modified team-based learning (mTBL) is designed for junior and senior emergency medicine and pediatric residents.

**Introduction/Background:**

Febrile seizures are the most common cause of seizures in children under 5 years old and are frequently evaluated in the emergency department.^1^,^2^ Febrile seizures can be frightening for parents to witness and often necessitate extensive parental reassurance and education by the emergency medicine (EM) provider. Most febrile seizures are brief, do not require a broad workup, and have a benign prognosis. With introduction of conjugate vaccines for *Haemophilus influenzae* type B (Hib) and *Streptococcus pneumoniae* in the United States in 1987 and 2000 respectively, the incidence of bacterial meningitis is low, but still present.^3^–^7^ The most recent American Academy of Pediatrics practice guidelines no longer recommend routine lumbar puncture on children presenting with simple febrile seizures.^2^ A review of the current literature shows that bacterial meningitis in children after a complex febrile seizure is unexpected when the clinical examination is not suggestive of meningitis or encephalitis.^5^–^8^ The goal of this mTBL is for residents to feel comfortable counseling parents about their child currently in the emergency department and the future risk of recurrence. The second goal is for residents to identify which patients presenting with fever and a seizure do require workup beyond simply identifying the source of the fever.

**Educational Objectives:**

By the end of this educational session, the learner will:

1. List the characteristics of a simple febrile seizure.
2. Discuss the management of a child with a simple vs. complex febrile seizure.
3. Discuss the risk factors that correlate with an increased risk of a subsequent febrile seizure.
4. Determine when a lumbar puncture should be considered in a febrile child with a seizure.
5. Identify when to give anti-epileptics and construct an algorithm for their use.
6. Discuss with parents, provide education and return precautions.

**Educational Methods:**

This didactic session is a mTBL. The classic learner responsible content (LRC) has been omitted and a short PowerPoint presentation is given to start the session before the individual and group readiness assessment tests.

**Research Methods:**

A post-TBL survey was given to each participant. A Likert scale was used to assess each participant’s assessment for the learning session in the following categories: overall, context, quality, and speaker feedback. They were also given fields to enter ways in which they would improve their practice after this learning exercise and suggestions they had for improving the current educational opportunity.

**Results:**

In the pilot session of this mTBL, 4 out of 11 participants (EM residents and pediatric emergency medicine [PEM] fellows) completed the post-TBL survey. Overall, this session was rated as “outstanding” (Likert 5/5) by 1 and “excellent” (Likert 4/5) by 3 for a weighted average of 4.25. All participants completing the survey found the activity “highly relevant,” “very engaging,” and wanted to repeat the activity in the future. Negative feedback consisted of wanting a video of a child having a seizure to be played and having a more interactive PowerPoint portion of the session like the interaction in the readiness assessment tests and group application exercise.

**Discussion:**

Overall the content was effective as evidenced by the list of ways residents said they would improve their practice on the post-TBL survey. In the future, I would extend the session from 60 minutes to 90 minutes to allow for more time for the group application exercise and discussion of answers. I found this to be an enjoyable, highly interactive experience with high engagement of the residents during the session.

**Topics:**

Simple febrile seizures, complex febrile seizures, seizure with fever, meningitis, lumbar puncture, status epilepticus.

## USER GUIDE

**Table:** 

List of Resources:	
Abstract	45
User Guide	47
Learner Materials	50
iRAT	50
gRAT	52
GAE	55
Instructor Materials	58
RAT Key	59
GAE Key	63

**Learner Audience:**

Interns, junior residents, senior residents, PEM fellows

**Time Required for Implementation:**

The preparation time for the instructor will likely be 60–90 minutes. The instructor should review and become familiar with the PowerPoint presentation they will be giving to start the educational session. Also, the instructor may find additional recommended reading helpful. Prior to the session, the instructor should prepare all of the copies of materials for the learners. There is no formal LRC for the learners, but 3 short overview references are included. Optional pre-reading would take no more than 10 minutes. The in-class portion is recommended to be 90 minutes.

**Recommended Number of Learners per Instructor:**

Ideally groups of 3–5 with mixed learner residency level in each group.

**Topics:**

Simple febrile seizures, complex febrile seizures, seizure with fever, meningitis, lumbar puncture, status epilepticus.

**Objectives:**

The goal of this mTBL is for residents to feel comfortable counseling parents about their child currently in the emergency department and the future risk of recurrence. The second goal is for residents to identify which patients presenting with fever and a seizure do require workup beyond identifying the source of the fever. By the end of this educational session, the learner will:

1. List the characteristics of a simple febrile seizure.
2. Discuss the management of a child with a simple vs. complex febrile seizure.
3. Discuss the risk factors that correlate with an increased risk of a subsequent febrile seizure.
4. Determine when a lumbar puncture should be considered in a febrile child with a seizure.
5. Identify when to give anti-epileptics and construct an algorithm for their use.
6. Discuss with parents, provide education and return precautions.

### Linked objectives and methods

Children with febrile seizures are commonly seen by residents in the emergency department (ED). A team-based learning activity helps them to differentiate what clinically should be done for evaluation and treatment of different types of seizures occurring with fever. The purpose of the PowerPoint presentation is to give the residents a framework to build their learning on; mainly to differentiate into categories the difference between a simple febrile seizure (SFS), a complex febrile seizure (CFS), and a seizure with fever. The first learning objective is met both in the readiness assessment test (RAT) question 1, and in case 1 of the group application exercise (GAE). Case 1 involves an infant with a SFS and case 2 an infant with a CFS. This allows the learner to compare and contrast the two scenarios, thus achieving objective 2. Objective 3 is met by completing RAT questions 3 and 4. Objective 4 is satisfied by RAT question 5 and the critical thinking pathway in the later portion of case 2. Case 2 is targeted to the more experienced learner, to recognize status epilepticus and initiate a management algorithm, achieving objective 5. Objective 6 empowers residents to synthesize the medical facts they have learned and apply them by educating and reassuring a child’s parents, as measured by the last portion of case 1. Group member discussion of RAT answers and GAE cases engages residents and solidifies new knowledge, while group discussion and review with the instructor probes critical thinking. This topic is well suited to a TBL activity because it allows the resident learner to move up Miller’s pyramid and Bloom’s taxonomy as the exercise progresses. The novice learner obtains foundational knowledge from the PowerPoint presentation, and as the RAT exercises and GAE cases evolve, learners are actively understanding and ultimately showing what they have learned in their written responses. By the end of this TBL, residents and fellows will have created a learning construct they can apply to their real patients in the ED.

### Recommended pre-reading for instructor

The instructor should become very familiar and comfortable with the included PowerPoint presentation slides, to be given by the instructor as the start of the TBL session. The instructor should also be familiar with all cases and instructional materials. Optional pre-reading includes:

- Patel N, Ram D, Swiderska N, et al. Clinical Review. Febrile seizures. *BMJ*. 2015;351:h4240. doi:10.1136/bmj.h4240.
- American Academy of Pediatrics, Subcommittee of Febrile Seizures. Neurodiagnostic evaluation of the child with a simple febrile seizure. *Pediatrics*. 2011;127:389–394. doi:10.1542/peds.2010-3318.
- Guedj R, Chappuy H, Titomanlio L, et al. Risk of bacterial meningitis in children 6 to 11 months of age with a first simple febrile seizure: a retrospective, cross-sectional, observational study. *Acad Emerg Med*. 2015;22:1290–1297. doi:10.1111/acem.12798.
- Guedj R, Chappuy H, Titomanlio L, et al. Do all children who present with a complex febrile seizure need a lumbar puncture? *Ann Emerg Med*. 2017;70(1):52–62. doi:10.1016/j.annemergmed.2016.11.024.

**Learner Responsible Content (LRC):** This mTBL has no true LRC because it is done during the first part of the session as a PowerPoint presented by the instructor. If the instructor would like to send out a topic overview for the learners, especially for the more novice learners such as interns and any medical students in attendance, any of the following reading is suggested:

- Koburov GT. Chapter 53: Seizures. In: Tenenbein M, Macias CG, eds. *Strange and Schafermeyer’s Pediatric Emergency Medicine*. 5th ed. New York, NY: McGraw-Hill, 2019:361–365.
- Kimia AA, Chiang VW. Chapter 57: Seizures. In: Shaw KN, Bachur RG, eds. *Fleisher and Ludwig’s Textbook of Pediatric Emergency Medicine*. 7th Ed. Philadelphia, PA: Wolters Kluwer, 2016:465–472.
- Liang T, Chao JH. Febrile seizures. In: Hoffman RJ, Wang VJ, eds. *Fleisher and Ludwig’s 5-Minute Pediatric Emergency Medicine Consult*. 2nd ed. Philadelphia, PA: Wolters Kluwer, 2020:292–293.
- Patel N, Ram D, Swiderska N, Mewasingh LD, Newton RW, Offringa M. Febrile seizures. *BMJ*. 2015;351:h4240. doi:10.1136/bmj.h4240.

### Results and tips for successful implementation

Prior to the session, the instructor should prepare materials:

- One individual readiness assessment test (iRAT) per learner.
- One group readiness assessment test (gRAT) per group (recommend 3–5 mixed residency level learners per group). Prepare the gRATs by making it an immediate feedback/assessment technique (IF/AT). You will need to purchase scratch-off stickers and prepare a gRAT IF/AT for each group. Cut the stickers so that they cover the letter choices on the gRAT. During the session, after each person completes their iRAT, the group will then collectively scratch off their answer and obtain immediate feedback. When the correct answer choice is scratched off, a star appears. When the incorrect answer is scratched-off the letter choice is reveled. The group can then try again by scratching-off another answer, until the correct answer, a star, is revealed. An example picture is shown in the gRAT section of this manuscript. The scratch-off stickers can be purchased on Amazon (https://www.amazon.com/My-Scratch-Offs-Rectangle-Sticker/dp/B00ENIB19Y). Website last checked April 28, 2020.
- One group application exercise (GAE) per learner (recommend the same groupings of residents for gRAT and GAE).
- Copies of all materials, including keys, for each instructor.

You will need approximately 90 minutes to conduct the learning session. I suggest the following time line:

1. PowerPoint lecture presented by instructor (15–20 minutes).
2. The instructor hands out the iRAT to all learners and has them complete it individually (5–10 minutes).
3. The instructor assigns learners into groups of 3–5. Ideally, each group mixed with junior and senior residents. Groups complete the gRAT (5–10 minutes).
4. The instructor will answer any questions related to the gRAT with all the groups participating (5–10 minutes).
5. Groups complete the GAE (20–25 minutes). All groups complete both cases; groups in close proximity to each other should start with opposite cases.
6. Review answers from the GAE (20–25 minutes). It is suggested that the instructor takes turns calling on groups to answer each question within the two cases. The instructor materials contain an explanation of answers for each question in the cases.

The pilot session was carried out March 11, 2020, at the start of severe acute respiratory syndrome coronavirus 2 (SARS-CoV-2) pandemic mitigation and safer at home practices in California. This likely explains why very few residents and fellows attended this education session. The pilot was tested on 11 learners. The results were obtained with a post-TBL survey using a Likert scale. In the pilot session of this mTBL, 4 out of 11 participants (EM residents and pediatric emergency medicine (PEM) fellows) completed the post-TBL survey. Overall, this session was rated as “outstanding” (Likert 5/5) by 1 and “excellent” (Likert 4/5) by 3 for a weighted average of 4.25. All participants completing the survey found the activity “highly relevant,” “very engaging,” and wanted to repeat the activity in the future. Negative feedback consisted of wanting a video of a child having a seizure to be played and having a more interactive PowerPoint portion of the session like the interaction in the readiness assessment tests and group application exercise. In the future, I would extend the session from 60 minutes to 90 minutes to allow for more time for the group application exercise and discussion of answers.

## Supplementary Information



## Figures and Tables

**Figure:**
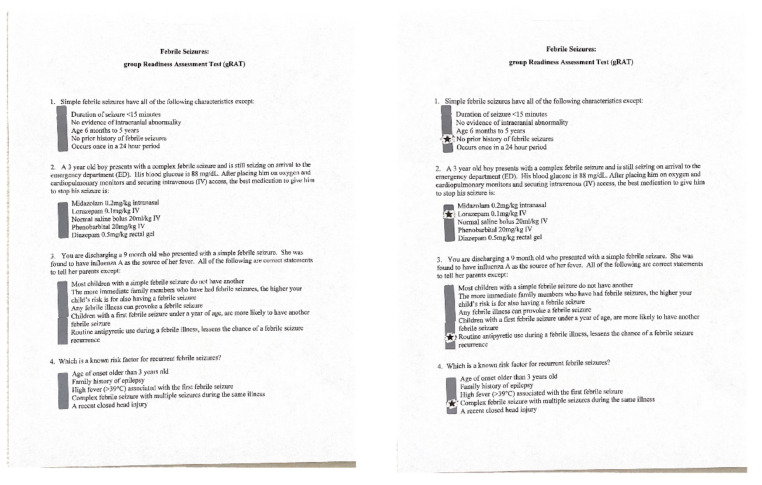


**Table:** 

Vital signs:	Temperature (T) 39.4°C	Heart rate (HR) 164	Blood pressure (BP) 99/70
Respiratory rate (RR) 34		Oxygen saturation (O_2_sat) 98% on room air	

**Table:** 

Leukocyte esterase	3+
Nitrite	Negative
Urine RBC	19/HPF
Urine WBC	>182/HPF
Urine bacteria	Moderate
Urine mucus	Many

**Table:** 

Vital signs:	T 40.4°C	HR 188	BP 136/89	RR 30	O_2_sat 100% on 15L mask

**Table:** 

Vital signs:	Temperature (T) 39.4°C	Heart rate (HR) 164	Blood pressure (BP) 99/70
Respiratory rate (RR) 34		Oxygen saturation (O_2_sat) 98% on room air	

**Table:** 

Leukocyte esterase	3+
Nitrite	Negative
Urine RBC	19/HPF
Urine WBC	>182/HPF
Urine bacteria	Moderate
Urine mucus	Many

**Table:** 

Vital signs:	T 40.4°C	HR 188	BP 136/89	RR 30	O_2_sat 100% on 15L mask

## LEARNER MATERIALS

### Febrile Seizures TBL: individual Readiness Assessment Test (iRAT)

1. Simple febrile seizures have all of the following characteristics except:
  - Duration of seizure <15 minutes
  - No evidence of intracranial abnormality
  - Age 6 months to 5 years
  - No prior history of febrile seizures
  - Occurs once in a 24-hour period
2. A 3-year-old boy presents with a complex febrile seizure and is still seizing on arrival to the emergency department (ED). His blood glucose is 88 mg/dL. After placing him on oxygen and cardiopulmonary monitors and securing intravenous (IV) access, the best medication to give him to stop his seizure is:
  - Midazolam 0.2mg/kg intranasal
  - Lorazepam 0.1mg/kg IV
  - Normal saline bolus 20ml/kg IV
  - Phenobarbital 20mg/kg IV
  - Diazepam 0.5mg/kg rectal gel
3. You are discharging a 9-month-old who presented with a simple febrile seizure. She was found to have influenza A as the source of her fever. All of the following are correct statements to tell her parents except:
  - Most children with a simple febrile seizure do not have another
  - The more immediate family members who have had febrile seizures, the higher your child’s risk is for also having a febrile seizure
  - Any febrile illness can provoke a febrile seizure
  - Children with a first febrile seizure under a year of age are more likely to have another febrile seizure
  - Routine antipyretic use during a febrile illness lessens the chance of a febrile seizure recurrence
4. Which is a known risk factor for recurrent febrile seizures?
  - Age of onset older than 3 years old
  - Family history of epilepsy
  - High fever (>39°C) associated with the first febrile seizure
  - Complex febrile seizure with multiple seizures during the same illness
  - A recent closed head injury
5. On which febrile patient should you consider doing a lumbar puncture?
  - 12-month-old female with a 2-minute generalized tonic-clonic (GTC) seizure with return to baseline.
  - 9-month-old male with 2-minute GTC seizure, currently on day 2 of antibiotics for a “throat infection.” Patient is behind on vaccines, having only had them at 2 months of age. No focal findings on exam, patient is sleepy but easily aroused.
  - 2-year-old female with 2 GTC seizures this afternoon, each 2–3 minutes, with return to baseline between seizures, and she is currently back to baseline.
  - 4-year-old male with reported 20-minute generalized seizure at home. He was postictal on arrival to ED which resolved within 30 minutes. He is now sitting coloring and eating in the ED.
  - 3-year-old female with 5-minute right arm shaking, stopped with single dose of intramuscular (IM) midazolam given by emergency medical services (EMS). On arrival to the ED she is sleepy but easily aroused. She has negative Kernig and Brudzinski signs. She has no focal neurological deficits.

### Febrile Seizures TBL: group Readiness Assessment Test (gRAT)

To make a gRAT using the immediate feedback/assessment technique (IF/AT), you will need to purchase scratch-off stickers and prepare a gRAT IF/AT for each group. The scratch-off stickers can be purchased on Amazon (https://www.amazon.com/My-Scratch-Offs-Rectangle-Sticker/dp/B00ENIB19Y). Website last checked April 28, 2020. Cut the stickers so that they cover the letter choices on the gRAT as demonstrated in the 1^st^ photo below. During the session, the group will then collectively scratch off their answer and obtain immediate feedback. When the correct answer choice is scratched off, a star appears, as demonstrated in the 2^nd^ photo below. When the incorrect answer is scratched-off, the letter choice is reveled. The group can then try again by scratching off another answer, until the correct answer, a star, is revealed.

1. Simple febrile seizures have all of the following characteristics except:
  a. Duration of seizure <15 minutes
  b. No evidence of intracranial abnormality
  c. Age 6 months to 5 years
  ★ d. No prior history of febrile seizures
  e. Occurs once in a 24-hour period
2. A 3-year-old boy presents with a complex febrile seizure and is still seizing on arrival to the emergency department (ED). His blood glucose is 88 mg/dL. After placing him on oxygen and cardiopulmonary monitors and securing intravenous (IV) access, the best medication to give him to stop his seizure is:
  a. Midazolam 0.2mg/kg intranasal
  ★ b. Lorazepam 0.1mg/kg IV
  c. Normal saline bolus 20ml/kg IV
  d. Phenobarbital 20mg/kg IV
  e. Diazepam 0.5mg/kg rectal gel
3. You are discharging a 9-month-old who presented with a simple febrile seizure. She was found to have influenza A as the source of her fever. All of the following are correct statements to tell her parents except:
  a. Most children with a simple febrile seizure do not have another
  b. The more immediate family members who have had febrile seizures, the higher your child’s risk is for also having a febrile seizure
  c. Any febrile illness can provoke a febrile seizure
  d. Children with a first febrile seizure under a year of age are more likely to have another febrile seizure
  ★ e. Routine antipyretic use during a febrile illness lessens the chance of a febrile seizure recurrence
4. Which is a known risk factor for recurrent febrile seizures?
  a. Age of onset older than 3 years old
  b. Family history of epilepsy
  c, High fever (>39°C) associated with the first febrile seizure
  ★ d. Complex febrile seizure with multiple seizures during the same illness
  e. A recent closed head injury
5. On which febrile patient should you consider doing a lumbar puncture?
  a. 12-month-old female with a 2-minute generalized tonic-clonic (GTC) seizure with return to baseline.
  ★ b. 9-month-old male with 2-minute GTC seizure, currently on day 2 of antibiotics for a “throat infection.” Patient is behind on vaccines, having only had them at 2 months of age. No focal findings on exam, patient is sleepy but easily aroused.
  c. 2-year-old female with 2 GTC seizures this afternoon, each 2–3 minutes, with return to baseline between seizures, and she is currently back to baseline.
  d. 4-year-old male with reported 20-minute generalized seizure at home. He was postictal on arrival to ED which resolved within 30 minutes. He is now sitting coloring and eating in the ED.
  e. 3-year-old female with 5-minute right arm shaking, stopped with single dose of intramuscular (IM) midazolam given by emergency medical services (EMS). On arrival to the ED she is sleepy but easily aroused. She has negative Kernig and Brudzinski signs. She has no focal neurological deficits.

### Febrile Seizures TBL: Group Application Exercise (GAE)

#### Case 1

A 10-month-old male is brought into the emergency department (ED) by emergency medical services (EMS). Per mom, he was noted to have “full body” shaking, staring, and was unresponsive. The episode lasted for 3 minutes. Mom also noted purple lips but states he was still breathing. After the seizure activity she described him as sleepy-appearing, serious and calm for about 20 minutes. On arrival to the ED he is back to his baseline. He developed a temperature of 99.3°F this morning with mild cough. He otherwise has had no other symptoms and was well yesterday. Mom gave acetaminophen once this morning for fever. He was born full term, is fully vaccinated, and has no significant past medical history. Blood glucose measured by EMS was 91 mg/dL. His physical exam shows a playful, interactive infant with no neurological deficits and no focal signs of bacterial infection.

1. What type of seizure did he have?
2. What imaging or studies, if any, would you order?
3. His catheterized urinalysis shows:
  What additionally will you order in the ED?
4. How will you disposition this patient?
5. When he is discharged home, what will you tell his mother regarding what to do at home if he has another seizure?

#### Case 2

A 14-month-old male presents to the ED with his mother around 5pm for 2 episodes of seizure activity at home. He began with a tactile fever yesterday and mild cough. While being triaged, he had a generalized tonic-clonic seizure (GTC) with his eyes rolling back. He was brought back to a room, placed on oxygen and a cardiopulmonary monitor, and an IV was placed. As lorazepam 0.1mg/kg IV was given, the seizing stopped. The seizure in the ED lasted about 5 minutes. Blood glucose 97 mg/dL. His examination after the seizure was notable for tachycardia, clear lungs with unlabored breathing, supple neck, not responding to painful stimuli, and no focal neurological deficits.

History of present illness: The first seizure occurred last night about 9pm and was described as generalized and lasted about 10 minutes. He was taken last night to an outside ED, examined and discharged home at his baseline with no testing. He vomited once after discharge home, but since tolerated milk. Today around 3pm mom witnessed a 2-minute GTC seizure, prompting her to bring him back to the ED. Per mom, between the seizures he returned to baseline and had been acting normally. His mom had been giving acetaminophen and ibuprofen at home.

Past medical history: Born full term, normal spontaneous vaginal delivery, pregnancy complicated by maternal methamphetamine use. In the NICU for 21 days for GBS bacteremia and +methamphetamine drug screen. No developmental delay. Patient with prior history of seizure and fever, 2 months ago, witnessed by daycare, generalized, duration unknown. Patient with additional 2-minute GTC seizure witnessed by mom 1 month ago, occurred with fever.

Social history: +daycare, lives with mom, no recent travel, vaccines up to date.

Family history: Paternal grandfather with seizures attributed to alcohol use. Mother with substance abuse and seizure related to drug use.

1. How would you classify his seizure?
2. What imaging or studies, if any, would you order?
3. His studies showed:
  Complete blood count (CBC): White blood cell count (WBC) 3.9 × 10^9^/L with 11% neutrophils, 47% lymphocytes, 34% monocytes. Absolute neutrophil count (ANC) 624/L.
  Complete metabolic panel: unremarkable except carbon dioxide 19 mmol/L.
  C-reactive protein: 13.9 mg/L.
  Urinalysis, urine drug screen and influenza all negative.
  Non-contrast head computed tomography (CT): No acute intracranial hemorrhage, mass effect, or hydrocephalus.
  What should you do next?
4. On re-examination (about 10 minutes after IV lorazepam was given) patient is noted to still be sleepy and is fussy. He is more responsive to painful stimuli and has no focal deficits. After he returns from head CT, you re-assess the patient (about 45 minutes after IV lorazepam) and he is now awake and alert, sitting in bed, tracking the examiner and drinking from his bottle. What should you do next?
5. What should you do if, when you went to re-assess the patient (10 min after IV lorazepam) he began to seize again? What medications should you give? What additional studies should you order?
6. How would you disposition the patient in question 4? The patient in question 5?
7. What medication (and in what route) could you give to a seizing patient requiring a benzodiazepine that does not have IV access?

Answer keys to all exercises with explanations, are on the following pages.

Learners: Please do not proceed.

### Febrile Seizures TBL: Readiness Assessment Test Key (RAT Key)

1. Simple febrile seizures have all of the following characteristics except:
  - Duration of seizure <15 minutes
  - No evidence of intracranial abnormality
  - Age 6 months to 5 years
  - **No prior history of febrile seizures**
  - Occurs once in a 24-hour period
  Explanation: Simple febrile seizures are characterized by: a child having a fever, seizure activity <15 minutes with no focality, and only 1 episode in 24 hours.^1^,^2^,^9^ This eliminates choices (a) and (e). The age range febrile seizures are typically seen in is 6 months to 5 years,^1^–^8^ eliminating choice (c). To be diagnosed with a febrile seizure you cannot have evidence of intracranial infection, intracranial structural abnormality, developmental delay, or a prior afebrile seizure.^1^,^11^ This eliminates choice (b). Choice (d) is the correct answer because prior febrile seizures do not preclude you from having a diagnosis of another febrile seizure.
2. A 3-year-old boy presents with a complex febrile seizure and is still seizing on arrival to the emergency department (ED). His blood glucose is 88 mg/dL. After placing him on oxygen and cardiopulmonary monitors and securing intravenous (IV) access, the best medication to give him to stop his seizure is:
  - Midazolam 0.2mg/kg intranasal
  - **Lorazepam 0.1mg/kg IV**
  - Normal saline bolus 20ml/kg IV
  - Phenobarbital 20mg/kg IV
  - Diazepam 0.5mg/kg rectal gel
  Explanation: The correct answer choice is (b) lorazepam 0.1mg/kg IV. Benzodiazepines are first line abortive therapy for seizures in children.^1^,^10^,^11^ All of the dose per kilogram options listed are the correct dose for his age. Lorazepam IV will work more quickly and last longer than intranasal midazolam choice (a). Rectal diazepam can be used when IV access is not easily obtainable, choice (e). Normal saline, choice (c), will not abort a seizure, but it may be given to help with hydration. An IV phenobarbital load, choice (d), is the drug of choice for a persistent seizure failing to respond to IV lorazepam in a young child (usually under 1 year of age). In children over the age of 1 year, IV fosphenytoin 20mg PE/kg is commonly given as the first-choice IV load for persistent seizure activity.
3. You are discharging a 9-month-old who presented with a simple febrile seizure. She was found to have influenza A as the source of her fever. All of the following are correct statements to tell her parents except:
  - Most children with a simple febrile seizure do not have another
  - The more immediate family members who have had febrile seizures, the higher your child’s risk is for also having a febrile seizure
  - Any febrile illness can provoke a febrile seizure
  - Children with a first febrile seizure under a year of age, are more likely to have another febrile seizure
  - **Routine antipyretic use during a febrile illness, lessens the chance of a febrile seizure recurrence**
  Explanation: Choice (e) is the only false statement. Randomized controlled trials have shown that antipyretic use does not prevent or lessen the chance of febrile seizures.^8^,^12^,^13^ It is important for parents to know this since they may blame themselves for not giving an antipyretic before their child had a febrile seizure. Antipyretics still should be given with the intent of helping the child to feel more comfortable. Choice (a) is true; 2/3 of children will not have a 2^nd^ febrile seizure.^2^,^11^ Choice (b) is true; family history of febrile seizures increases a 1^st^ degree relative’s risk of also having a febrile seizure.^1^,^11^ General prevalence risk for febrile seizures is 2–5%.^1^,^2^,^4^–^8^ This increases to ~20% when one sibling is affected and ~33% increased risk when both parents and a sibling have had a febrile seizure.^1^,^11^ Choice (c) is correct; any illness that causes a fever can lead to a febrile seizure; respiratory illnesses are most common. Choice (d) is also correct; having a 1^st^ febrile seizure when <1 year old, a child has a 50% probability of a recurrent febrile seizure, whereas having a 1^st^ febrile seizure when >1 year, a child has a 30% probability of recurrence.^1^,^11^
4. Which is a known risk factor for recurrent febrile seizures?
  - Age of onset older than 3 years old
  - Family history of epilepsy
  - High fever (>39°C) associated with the first febrile seizure
  - **Complex febrile seizure with multiple seizures during the same illness**
  - A recent closed head injury
  Explanation: Risk factors for increased risk of a recurrent febrile seizure are:^1^,^11^
  - Young age at 1^st^ febrile seizure (<1 year old)
  - Family history of febrile seizure in a 1^st^ degree relative
  - Relatively low-grade fever (< 39°C) with febrile seizure
  - Shorter duration (<1hr) of fever before seizure
  - Multiple seizures during the same febrile illness
  - Daycare attendance (Possibly due to increased viral exposure therefore increased risk of fever)
  The more risk factors that are present, the higher the chance for recurrence. With no risk factors present there is a ~4% chance of recurrent febrile seizure. With all of the above risk factors present the risk for recurrence increases to 80%.^1^ Choice (d) is the correct answer. Choice (a) is incorrect because this age is not high risk. Choice (b) is incorrect because family history of epilepsy has not consistently been associated with increased risk of recurrent febrile seizures. Choice (c) is incorrect because a relatively lower temperature (<39°C) is associated with recurrence. Choice (e) is incorrect because prior trauma does not increase the risk of a febrile seizure. In fact, a significant head trauma, resulting in structural damage, would preclude the diagnosis of a febrile seizure. The child could have a fever with seizure.
5. On which febrile patient should you consider doing a lumbar puncture?
  - 12-month-old female with a 2 minute generalized tonic-clonic (GTC) seizure with return to baseline.
  - **9-month-old male with 2-minute GTC seizure, currently on day 2 of antibiotics for a “throat infection.” Patient is behind on vaccines, having only had them at 2 months of age. No focal findings on exam, patient is sleepy but easily aroused**.
  - 2-year-old female with 2 GTC seizures this afternoon, each 2–3 minutes, with return to baseline between seizures, and she is currently back to baseline.
  - 4-year-old male with reported 20-minute generalized seizure at home. He was postictal on arrival to ED which resolved within 30 minutes. He is now sitting coloring and eating in the ED.
  - 3-year-old female with 5 minute right arm shaking, stopped with single dose of intramuscular (IM) midazolam given by emergency medical services (EMS). On arrival to the ED she is sleepy but easily aroused. She has negative Kernig and Brudzinski signs. She has no focal neurological deficits.
  Explanation: The patient in choice (a) had a simple febrile seizure; no lumbar puncture (LP) is needed. The patients in choice (c) and (d) had complex febrile seizures and are back to baseline; no LP is needed. The patient in choice (e) had a focal complex seizure, and has no neurologic findings other than being postictal. It would be appropriate to observe her in the ED; if the postictal period clears within an hour and she is at her baseline, no LP is needed. If she continues to be postictal or her exam changes, a LP should be done at that time. Choice (b) is the best answer. This patient is both not fully vaccinated with his primary series (incomplete Hib and *Streptococcus pneumoniae* vaccination increases the risk of bacterial meningitis) and has been pre-treated with antibiotics, which could incompletely treat and mask meningitis.^1^,^14^

### Febrile Seizures TBL: Group Application Exercise (GAE) Key

#### Case 1

A 10-month-old male is brought into the emergency department (ED) by emergency medical services (EMS). Per mom, he was noted to have “full body” shaking, staring, and was unresponsive. The episode lasted for 3 minutes. Mom also noted purple lips but states he was still breathing. After the seizure activity she described him as sleepy-appearing, serious and calm for about 20 minutes. On arrival to the ED he is back to his baseline. He developed a temperature of 99.3°F this morning with mild cough. He otherwise has had no other symptoms and was well yesterday. Mom gave acetaminophen once this morning for fever. He was born full term, is fully vaccinated, and has no significant past medical history. Blood glucose measured by EMS was 91 mg/dL. His physical exam shows a playful, interactive infant with no neurological deficits and no focal signs of bacterial infection.

1. What type of seizure did he have?
  He had a simple febrile seizure (SFS). He is vaccinated, developmentally normal, without signs of central nervous system (CNS) infection, and is back to baseline in the ED. Criteria for SFS are:^1^,^2^,^9^
  - Fever (temp ≥38°C by any method)
  - No evidence of intracranial infection, developmental delay, intracranial abnormality, toxin, or prior history of afebrile seizure
  - Age 6 months-5 years
  - Generalized
  - <15-minute duration (usually lasts <2–3 minutes)
  - Occurs once in 24 hours
2. What imaging or studies, if any, would you order?
  In SFS it is important to limit interventions. Studies should only be ordered as needed to evaluate for the source of the fever. Routine blood work, lumbar puncture (LP) and head imaging are not necessary and should not be ordered. The incidence of bacteremia in febrile kids <24 months with or without a febrile seizure is the same.^2^
3. His catheterized urinalysis shows:
  What additionally will you order in the ED?
  Given the patient’s age, appropriate studies would be catheterized urinalysis and culture and depending on the time of year possibly viral studies such as influenza. He has a urinary tract infection. Antipyretics should be given to reduce the temperature and provide comfort. A single dose of intravenous (IV) or intramuscular (IM) antibiotics, such as Ceftriaxone, and a prescription for oral antibiotics, would be appropriate for treating pyelonephritis. Blood work is not needed because of the seizure, but may be ordered at the physician’s preference for evaluation of pyelonephritis. The incidence of bacteremia in febrile kids <24 months with or without a febrile seizure is the same.^2^
4. How will you disposition this patient?
  As long as he is not vomiting and able to tolerate his home oral antibiotics, he may be discharged home with close follow up with his primary medical doctor (PMD).
5. When he is discharged home, what will you tell his mother regarding what to do at home if he has another seizure?
  Acknowledge that febrile seizures are frightening for parents to witness. Reiterate that they do not cause brain damage, paralysis, or any other permanent problem.^11^ Talk with the mother, explaining that if he has another seizure she should:
  - Gently lower child to the floor on his side to prevent choking
  - Don’t put your fingers in his mouth
  - Don’t try and give antipyretics until the seizure is over and your child is fully awake
  - Call 911 if seizure lasts 5 minutes or greater, your child becomes blue in the face, or has trouble breathing
  - Return to the ED if your child has another seizure within 24 hours of the first seizure

#### Case 2

A 14-month-old male presents to the ED with his mother around 5pm for 2 episodes of seizure activity at home. He began with a tactile fever yesterday and mild cough. While being triaged, he had a generalized tonic-clonic seizure (GTC) with his eyes rolling back. He was brought back to a room, placed on oxygen and a cardiopulmonary monitor, and an IV was placed. As lorazepam 0.1mg/kg IV was given, the seizing stopped. The seizure in the ED lasted about 5 minutes. Blood glucose 97 mg/dL. His examination after the seizure was notable for tachycardia, clear lungs with unlabored breathing, supple neck, not responding to painful stimuli, and no focal neurological deficits.

History of present illness: The first seizure occurred last night about 9pm and was described as generalized and lasted about 10 minutes. He was taken last night to an outside ED, examined and discharged home at his baseline with no testing. He vomited once after discharge home, but since tolerated milk. Today around 3pm mom witnessed a 2-minute GTC seizure, prompting her to bring him back to the ED. Per mom, between the seizures he returned to baseline and had been acting normally. His mom had been giving acetaminophen and ibuprofen at home.

Past medical history: Born full term, normal spontaneous vaginal delivery, pregnancy complicated by maternal methamphetamine use. In the NICU for 21 days for GBS bacteremia and +methamphetamine drug screen. No developmental delay. Patient with prior history of seizure and fever, 2 months ago, witnessed by daycare, generalized, duration unknown. Patient with additional 2-minute GTC seizure witnessed by mom 1 month ago, occurred with fever.

Social history: +daycare, lives with mom, no recent travel, vaccines up to date.

Family history: Paternal grandfather with seizures attributed to alcohol use. Mother with substance abuse and seizure related to drug use.

1. How would you classify his seizure?
  This is a complex febrile seizure (CFS). He has had multiple episodes within 24 hours. To be classified as a CFS a child will have any 1 (or more) of the following features: seizure duration >15 minutes, >1 episode in 24 hours, or focal nature of seizure at any time.^1^,^2^,^9^
  To be classified as a febrile seizure, a child cannot have any evidence of intracranial infection, intracranial structural abnormality, development delay, toxin, or prior afebrile seizure.^1^,^11^
2. What imaging or studies, if any, would you order?
  Labs and imaging should be ordered to evaluate source of fever. Given numerous episodes of seizure during this illness, and he is not currently back to baseline, screening labs and head CT would be appropriate. LP should be considered if he has a prolonged postictal period (>1 hour).^5^–^8^
3. His studies showed:
  Complete blood count (CBC): White blood cell count (WBC) 3.9 ×10^9^/L with 11% neutrophils, 47% lymphocytes, 34% monocytes. Absolute neutrophil count (ANC) 624/L.
  Complete metabolic panel: unremarkable except carbon dioxide 19 mmol/L.
  C-reactive protein: 13.9 mg/L.
  Urinalysis, urine drug screen and influenza all negative.
  Non-contrast head computed tomography (CT): No acute intracranial hemorrhage, mass effect, or hydrocephalus.
  What should you do next?
  Labs show that he is mildly dehydrated, carbon dioxide is low, and he is neutropenic, ANC of 624. IV fluids and empiric Ceftriaxone should be considered. The next step should be to reassess your patient with a thorough repeat neurological examination to determine if an LP is needed.
4. On re-examination (about 10 minutes after IV lorazepam was given) patient is noted to still be sleepy and is fussy. He is more responsive to painful stimuli and has no focal deficits. After he returns from head CT, you re-assess the patient (about 45 minutes after IV lorazepam) and he is now awake and alert, sitting in bed, tracking the examiner and drinking from his bottle. What should you do next?
  This patient is now back to baseline and with no signs of CNS infection, a LP is not indicated. Calling a neurology consult would be appropriate. Given his prior history of recurrent febrile seizures and this one being a CFS with multiple episodes, this patient is higher risk to have additional recurrent febrile seizures in the future. He also has a 6–8% chance of developing epilepsy.^8^,^11^ For this specific patient, neurology recommended admission for sleep-deprived electroencephalogram (EEG). His EEG was normal without epileptiform discharges, and he was discharged home to follow up with neurology as an outpatient; no antiepileptic medications were started.
5. What should you do if, when you went to re-assess the patient (10 min after IV lorazepam) he began to seize again? What medications should you give? What additional studies should you order?
  If he seized again, he should be treated as febrile status epileptics. Another dose of IV lorazepam (0.1mg/kg) should be given along with an IV loading dose of antiepileptic. IV phenobarbital and IV fosphenytoin are common first choices.
  IV phenobarbital is typically given in children <1 year of age and is the drug of choice for neonatal seizures. The dose is 20mg/kg IV load. It reaches peak brain levels in 10–20 minutes and it has a prolonged duration of action >48 hours. A post load level is commonly checked 3 hours after given, to help with inpatient management. The common side effects are respiratory depression (additive with benzodiazepines), sedation, and occasionally hypotension.^10^,^15^
  IV fosphenytoin is typically the drug of choice for older children. The dose is 20mg phenytoin equivalent (PE)/kg IV load; it is dosed in phenytoin equivalents. Peak brain levels are reached in 10–30 minutes. A post load level is also commonly checked 3 hours after given, to help with inpatient management. Common side effects are pruritus and paresthesia of the groin.^10^ Since this IV solution doesn’t contain propylene glycol (like phenytoin does) it can be given IM. The IM dose is the same, but the peak brain levels take much longer, 3 hours.
  If after an IV load with phenobarbital or fosphenytoin, the patient continues to seize, there are many options of the next medication choice, and a neurology consult to help with antiepileptic choice and management is appropriate. Commonly, a second load is given: either of the medication previously not given at full dose, or a second load of the medication already given at a half dose. If the patient continues to seize despite this, an IV drip is often needed. Medications that can be used for this are pentobarbital, diazepam, midazolam, or IV propofol.^10^ The IV infusion is titrated until there is burst suppression seen on EEG. This patient should also have a LP to evaluate for meningitis.
6. How would you disposition the patient in question 4? The patient in question 5?
  The patient in question 4 should have a neurology consult and recommendations for admission. The patient in question 5 should have a neurology consult for recommendations on additional mediations to control status epilepticus and to facilitate EEG placement. Patient 5 needs to be admitted to the pediatric intensive care unit.
7. What medication (and in what route) could you give to a seizing patient requiring a benzodiazepine that does not have IV access?
  If IV access is lost or unable to obtained, the following can be given:^10^
  - Diazepam
    ○ IV form given per rectum
      ▪ Onset 5–10 minutes
      ▪ 1^st^ dose: 0.5mg/kg, additional doses 0.25mg/kg, Max 20mg
    ○ Rectal gel (Diastat R)
      ▪ Dose is different for different age groups!
        - 0.5mg/kg for 2–5 years old
        - 0.3mg/kg for 6–11 years old
        - 0.2mg/kg for ≥12 years old
      ▪ Available in premeasured sizes: 2.5mg, 10mg (10mg AcuDial™ has ability to dial in 5mg, 7.5mg, or 10mg dose)
  - Midazolam
    ○ IM midazolam
      ▪ Onset ~15 minutes
      ▪ Dose 0.1mg/kg
    ○ Intranasal (IN) midazolam
      ▪ Onset 5–10 minutes
      ▪ Dose 0.2mg/kg
  - Intraosseous (IO) – Any IV medication can be given, same dose as IV route
