# Supplementary material for: Febrile Seizure Team-based Learning
Source: J Educ Teach Emerg Med. 2020 Oct 15;5(4):T45–68. doi: 10.21980/J8JD12 (PMC10334448; doi:10.21980/J8JD12)
Supplement: Supplementary file 1 [file jetem-5-4-t45-supp1.pptx]

## Slide 1
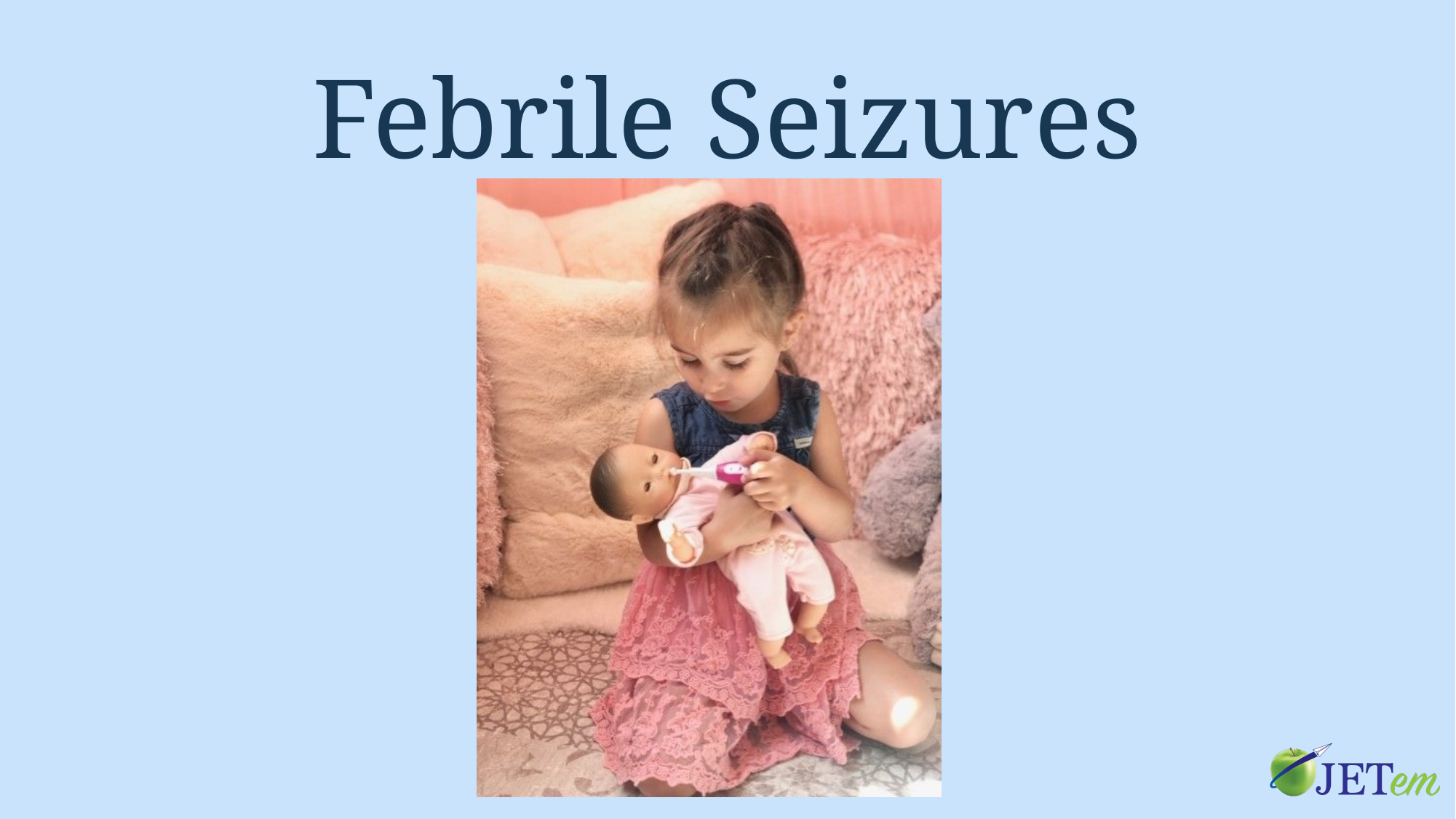

# Febrile Seizures

## Slide 2
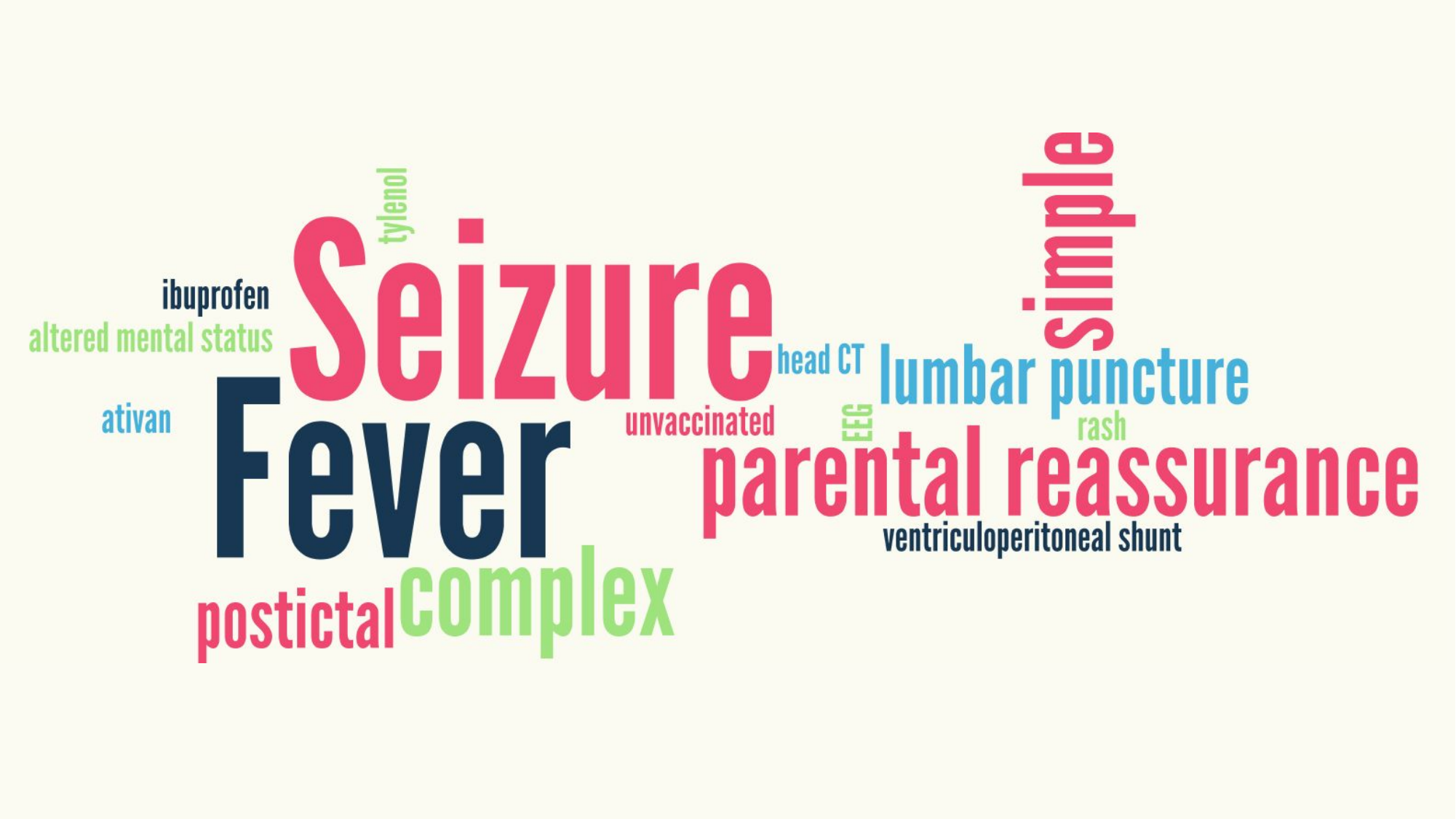

## Slide 3
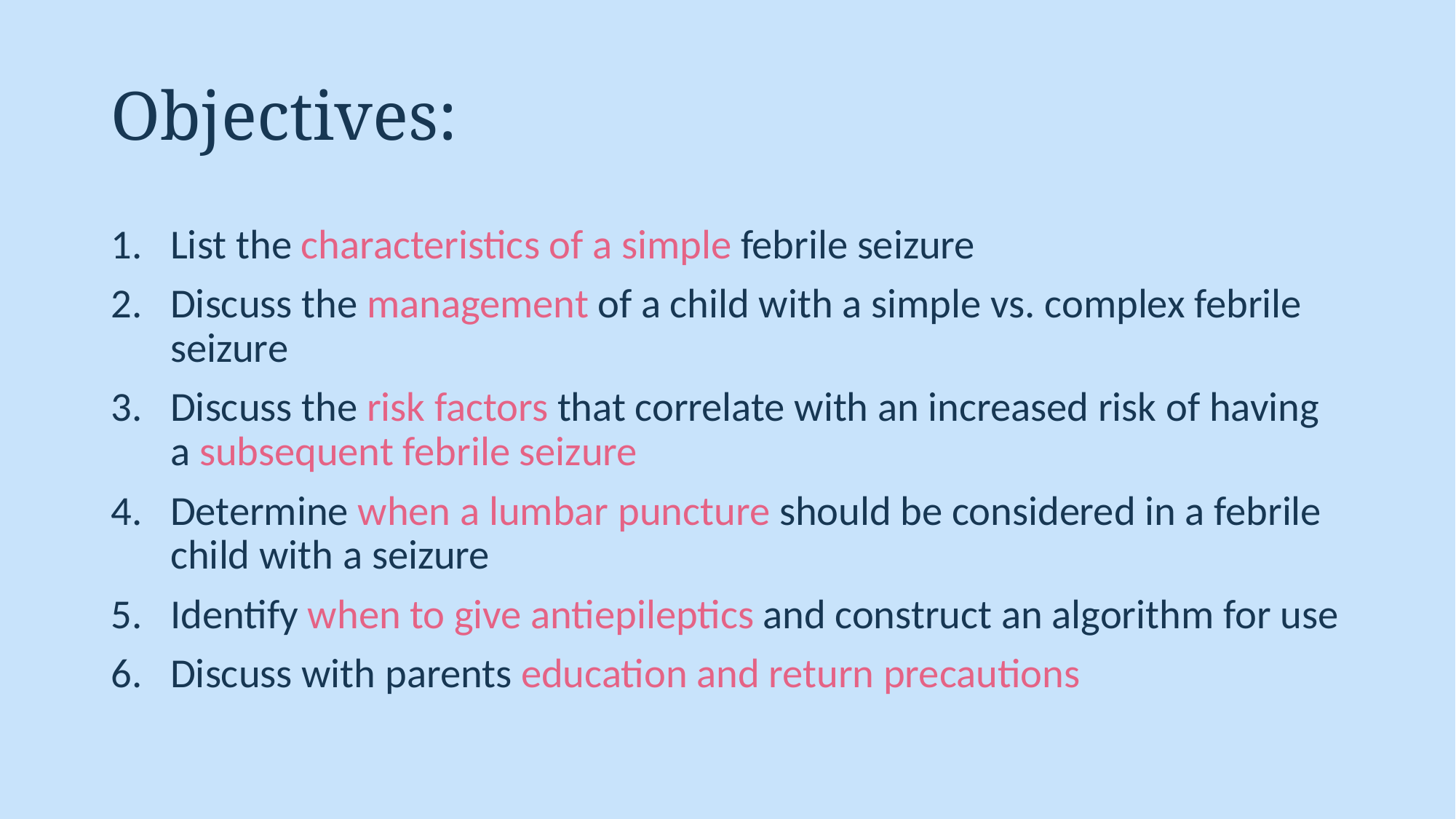

# Objectives:
List the characteristics of a simple febrile seizure
Discuss the management of a child with a simple vs. complex febrile seizure
Discuss the risk factors that correlate with an increased risk of having a subsequent febrile seizure
Determine when a lumbar puncture should be considered in a febrile child with a seizure
Identify when to give antiepileptics and construct an algorithm for use
Discuss with parents education and return precautions

## Slide 4
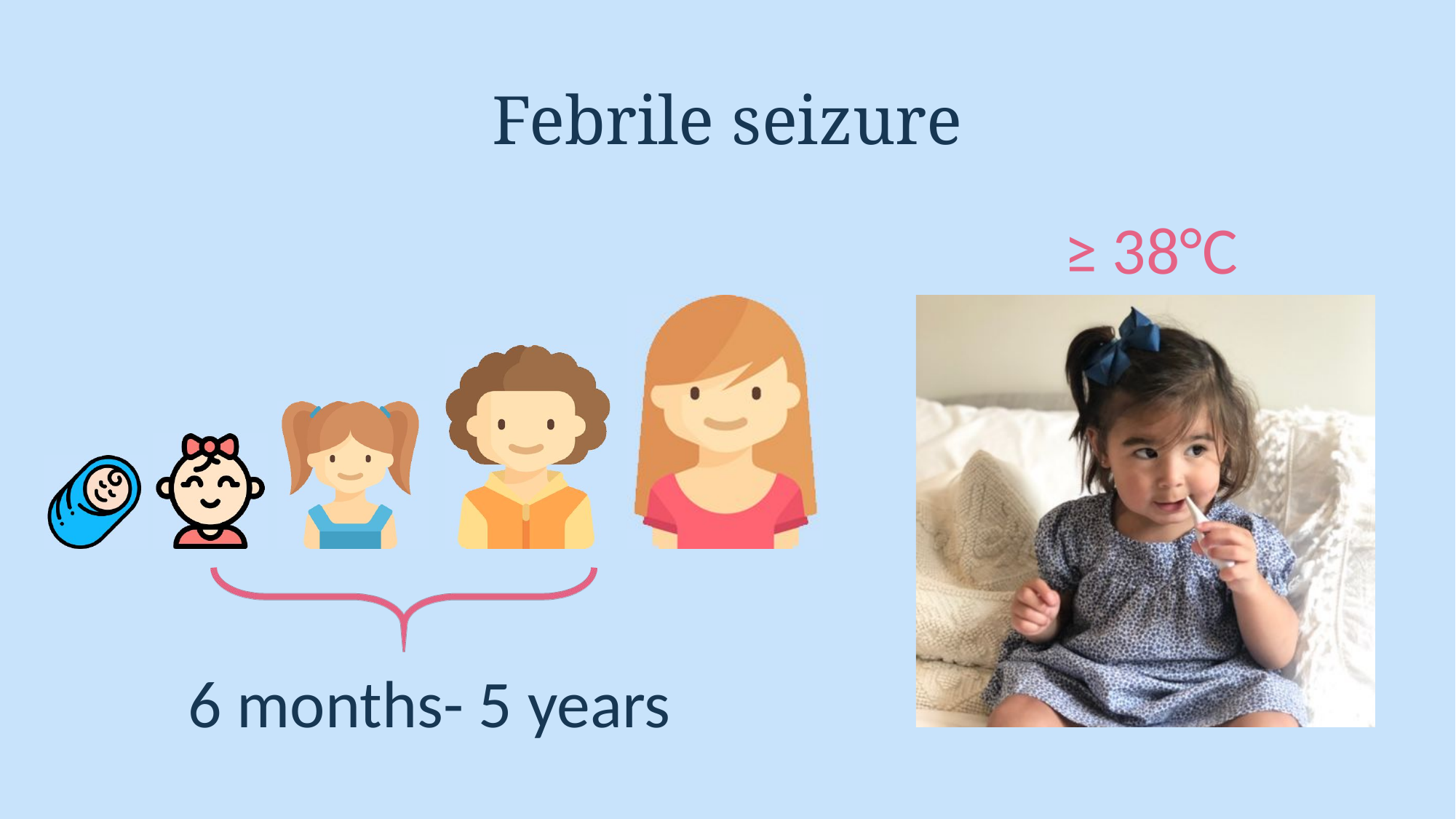

# Febrile seizure
≥ 38°C
6 months- 5 years

## Slide 5
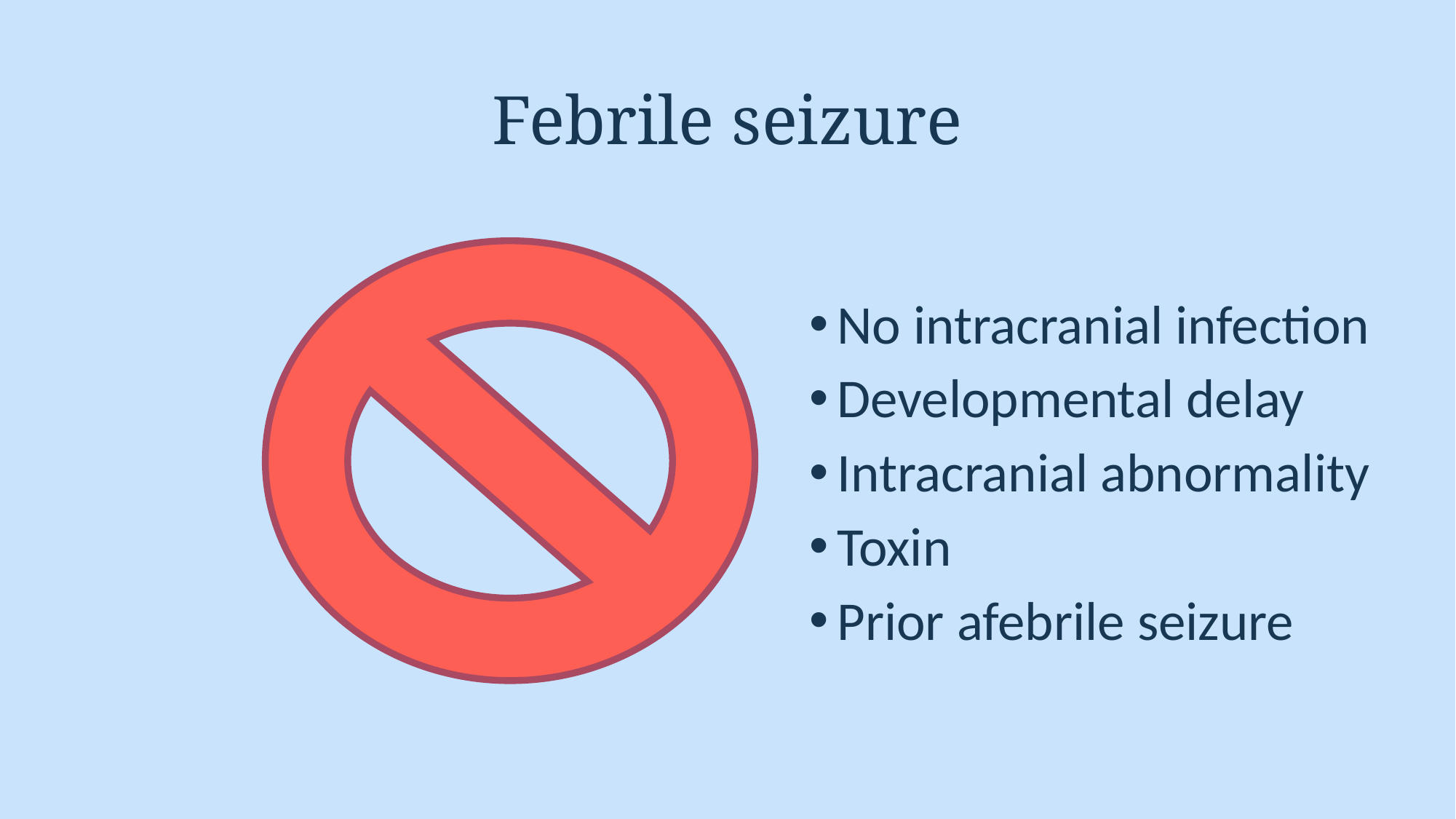

# Febrile seizure
No intracranial infection
Developmental delay
Intracranial abnormality
Toxin
Prior afebrile seizure

## Slide 6
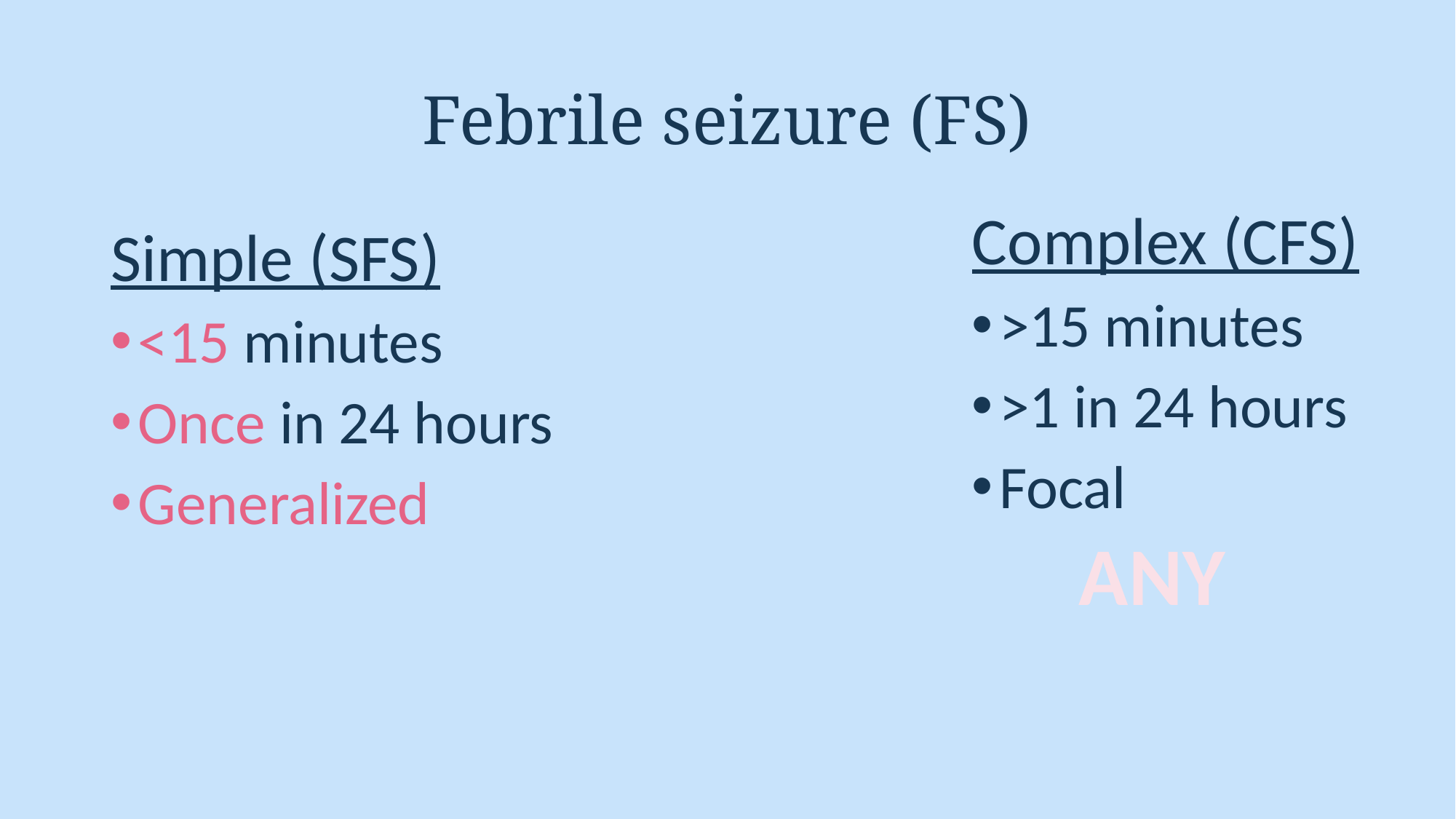

# Febrile seizure (FS)
Complex (CFS)
>15 minutes
>1 in 24 hours
Focal
Simple (SFS)
<15 minutes
Once in 24 hours
Generalized
ANY

## Slide 7
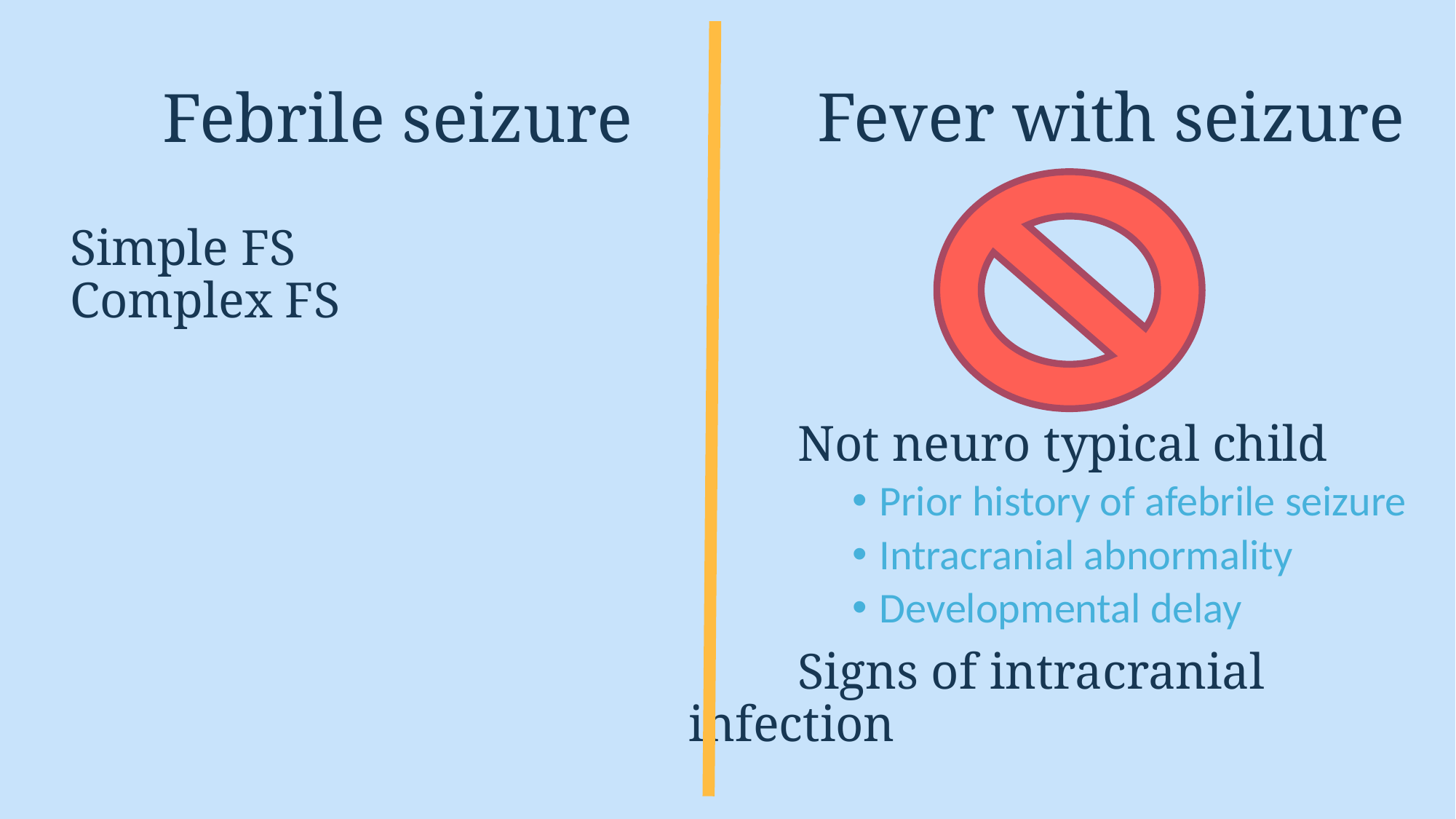

# Febrile seizure
Fever with seizure
Simple FS		Complex FS
	Not neuro typical child
Prior history of afebrile seizure
Intracranial abnormality
Developmental delay
	Signs of intracranial infection

## Slide 8
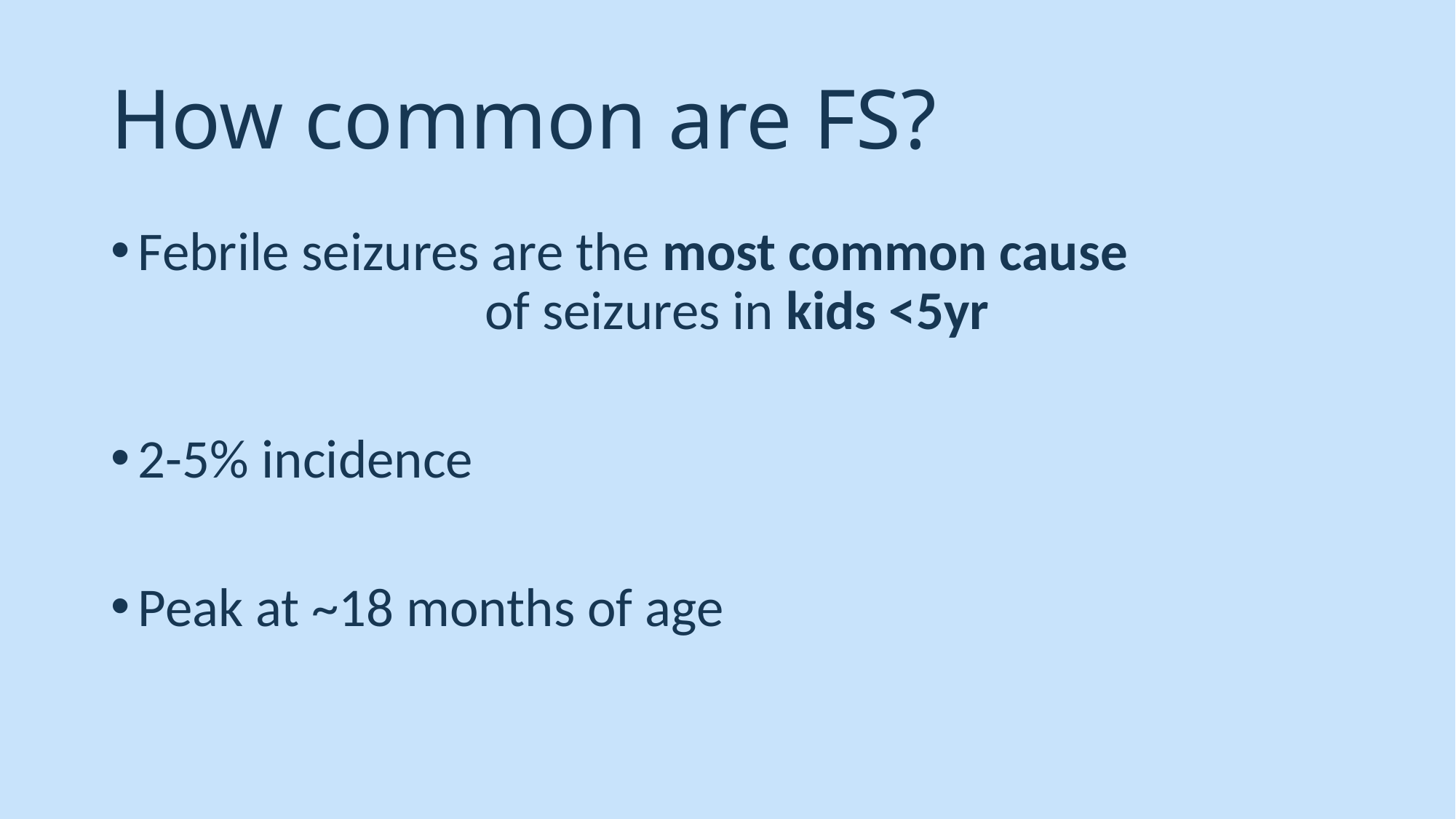

# How common are FS?
Febrile seizures are the most common cause of seizures in kids <5yr
2-5% incidence
Peak at ~18 months of age

## Slide 9
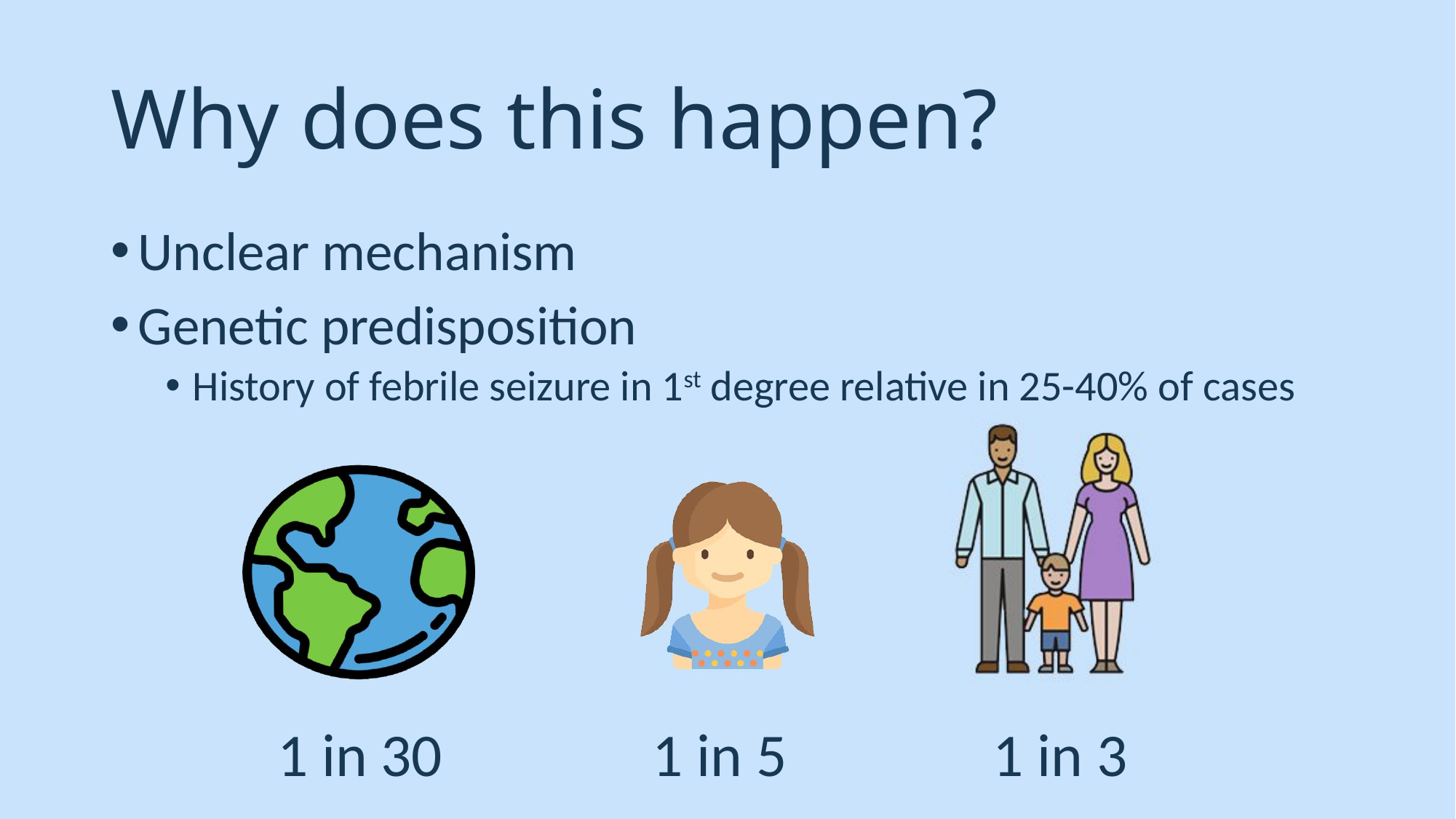

# Why does this happen?
Unclear mechanism
Genetic predisposition
History of febrile seizure in 1st degree relative in 25-40% of cases
1 in 30
1 in 5
1 in 3

## Slide 10
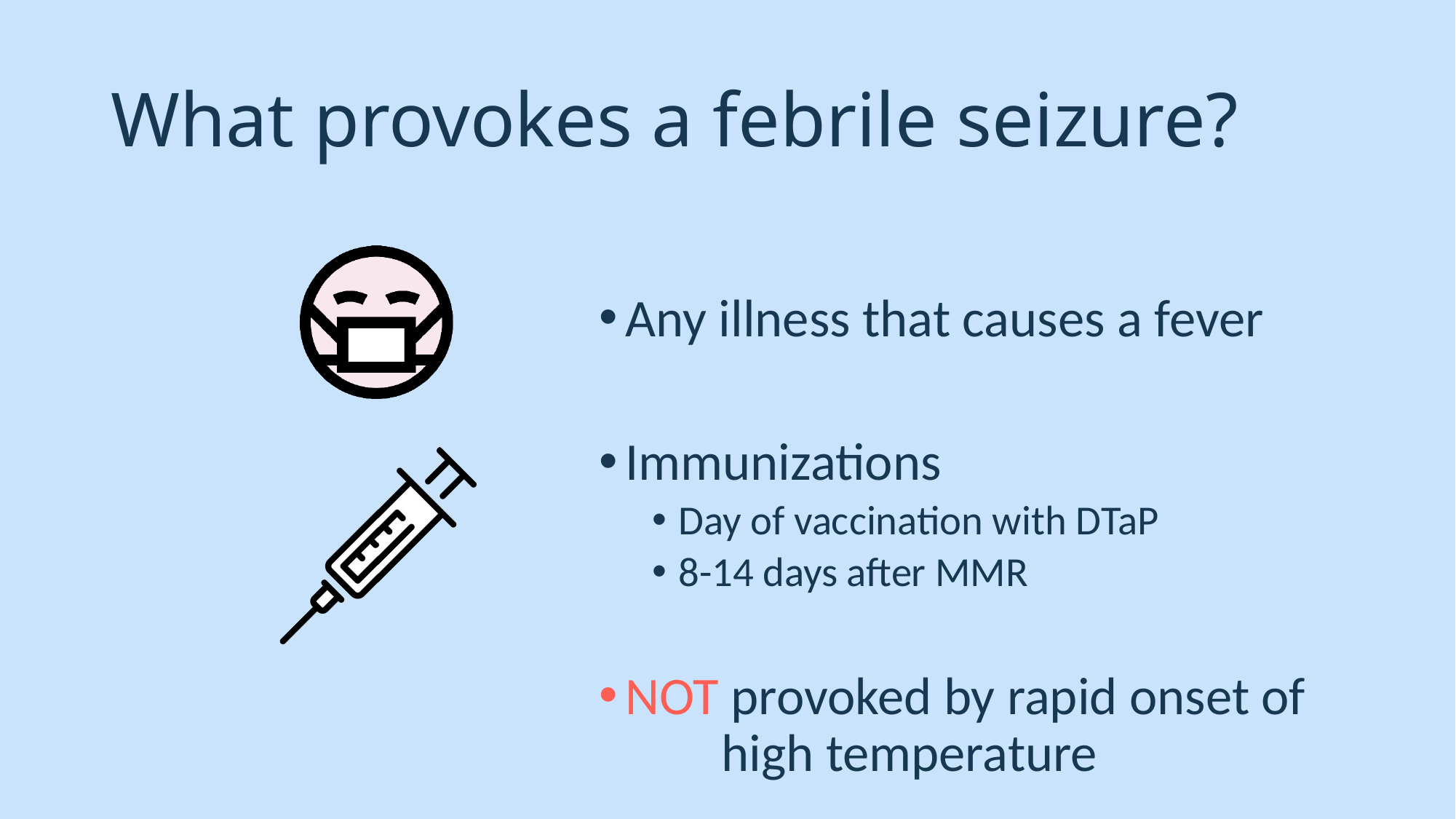

# What provokes a febrile seizure?
Any illness that causes a fever
Immunizations
Day of vaccination with DTaP
8-14 days after MMR
NOT provoked by rapid onset of high temperature

## Slide 11
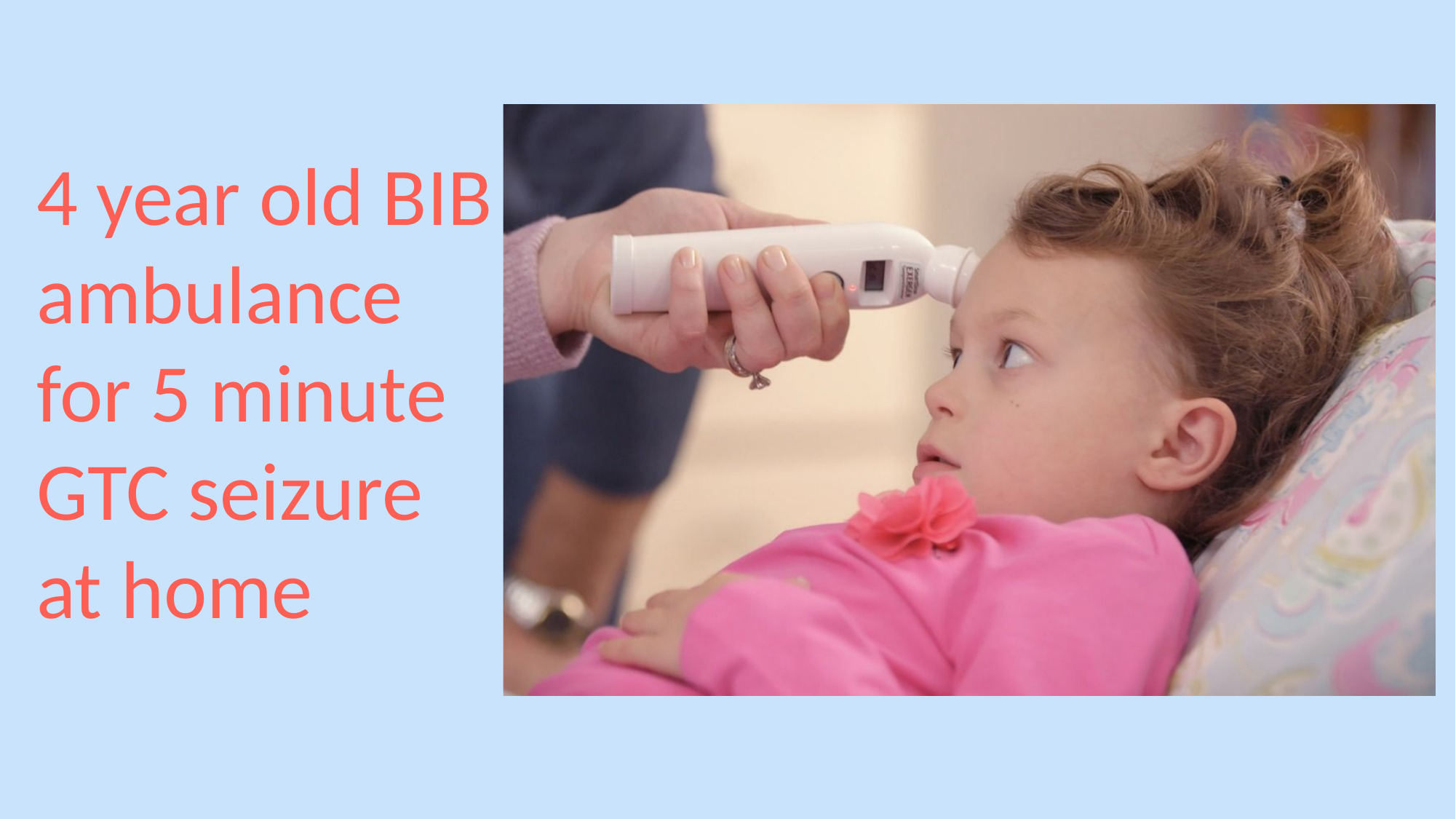

4 year old BIB ambulance for 5 minute GTC seizure at home

## Slide 12
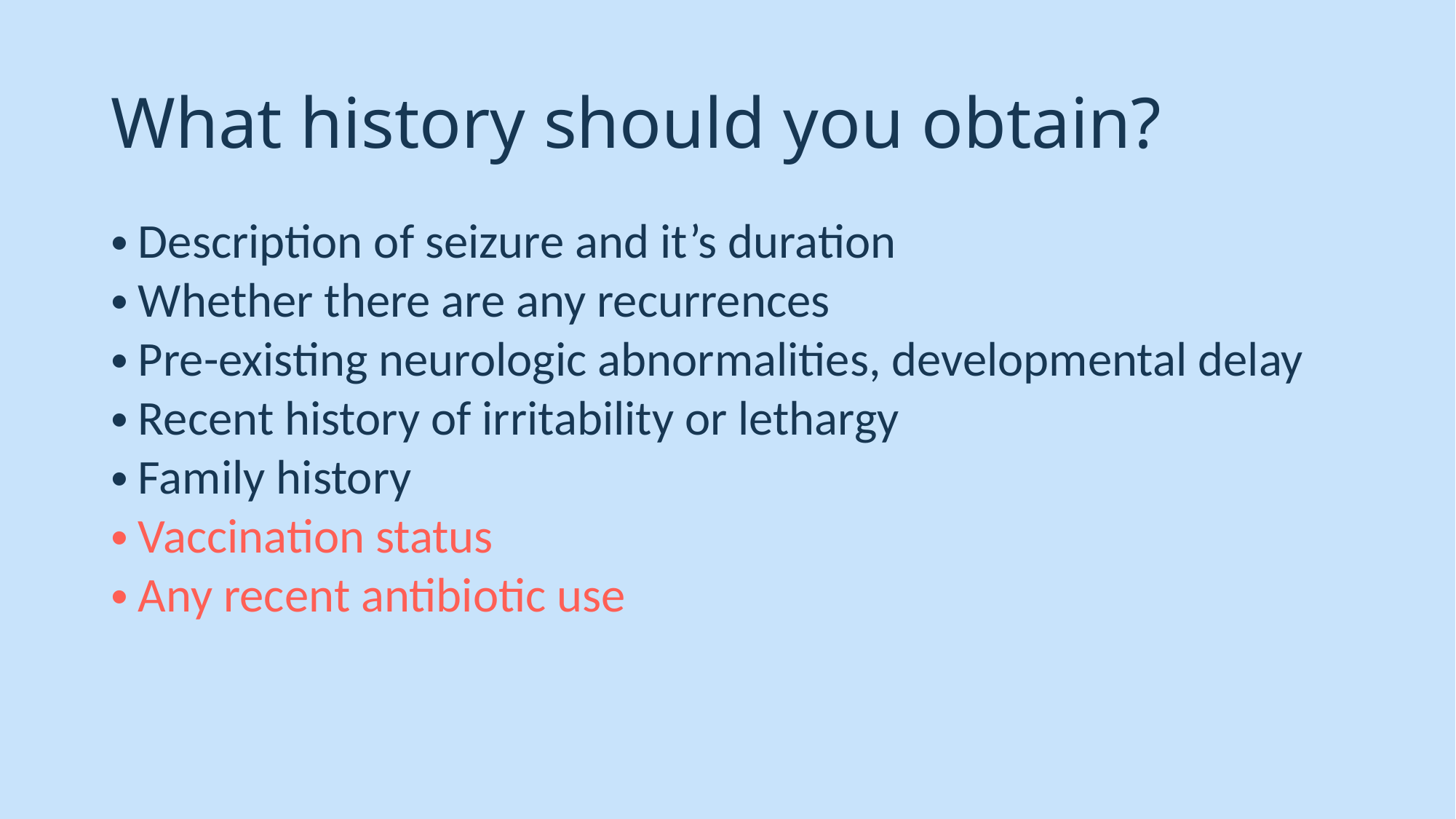

# What history should you obtain?
Description of seizure and it’s duration
Whether there are any recurrences
Pre-existing neurologic abnormalities, developmental delay
Recent history of irritability or lethargy
Family history
Vaccination status
Any recent antibiotic use

## Slide 13
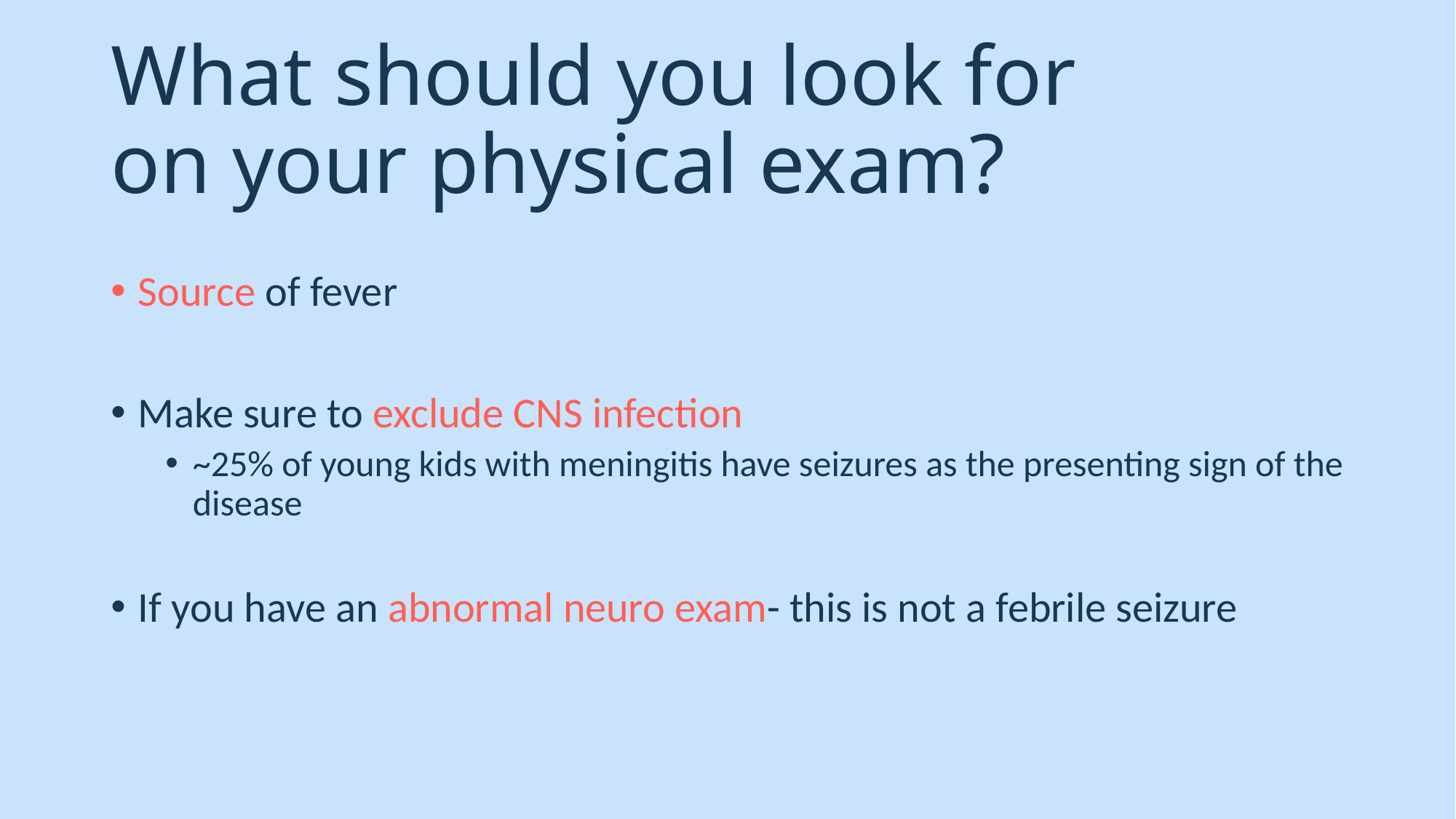

# What should you look for on your physical exam?
Source of fever
Make sure to exclude CNS infection
~25% of young kids with meningitis have seizures as the presenting sign of the disease
If you have an abnormal neuro exam- this is not a febrile seizure

## Slide 14
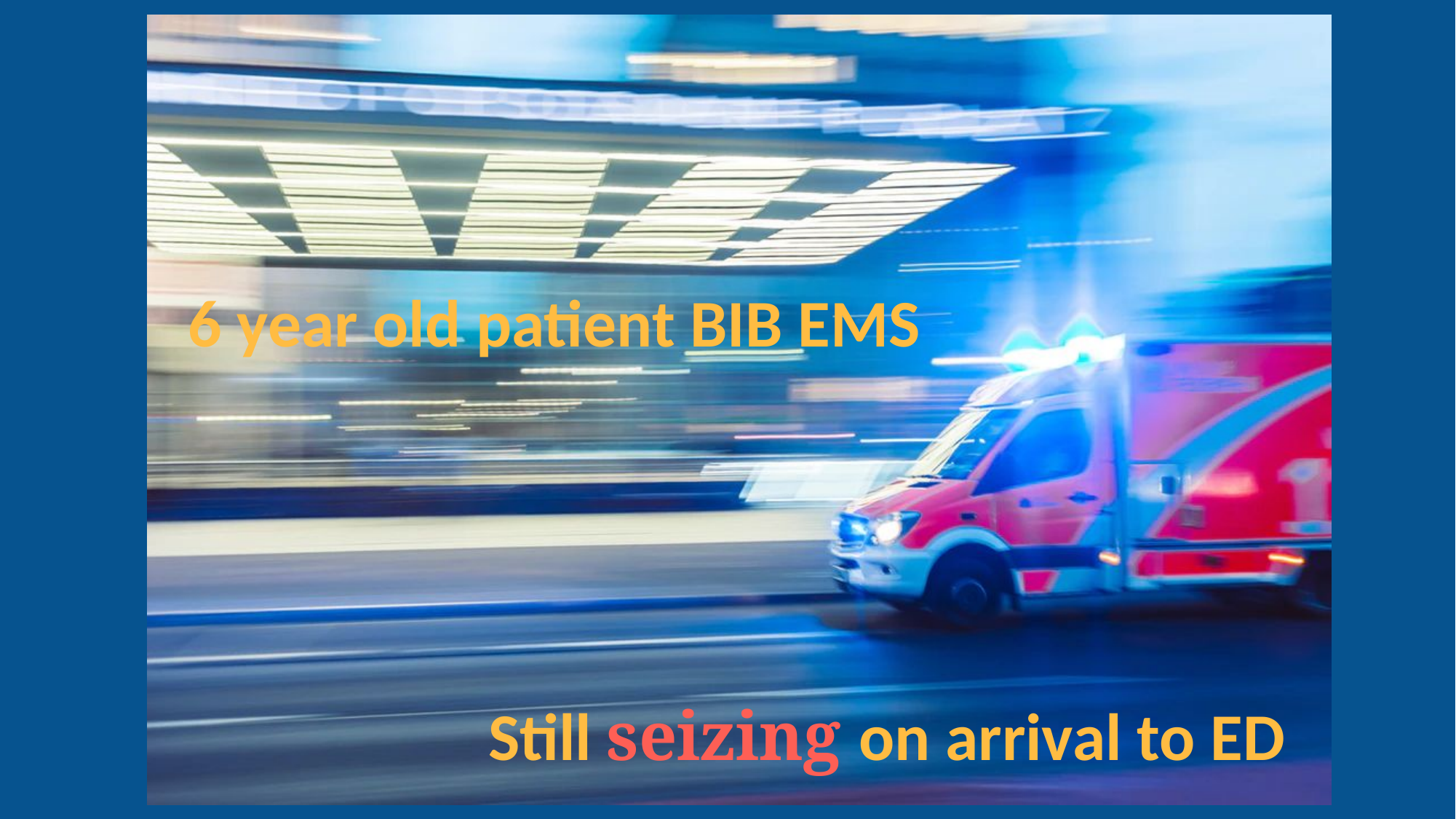

6 year old patient BIB EMS
Still seizing on arrival to ED

## Slide 15
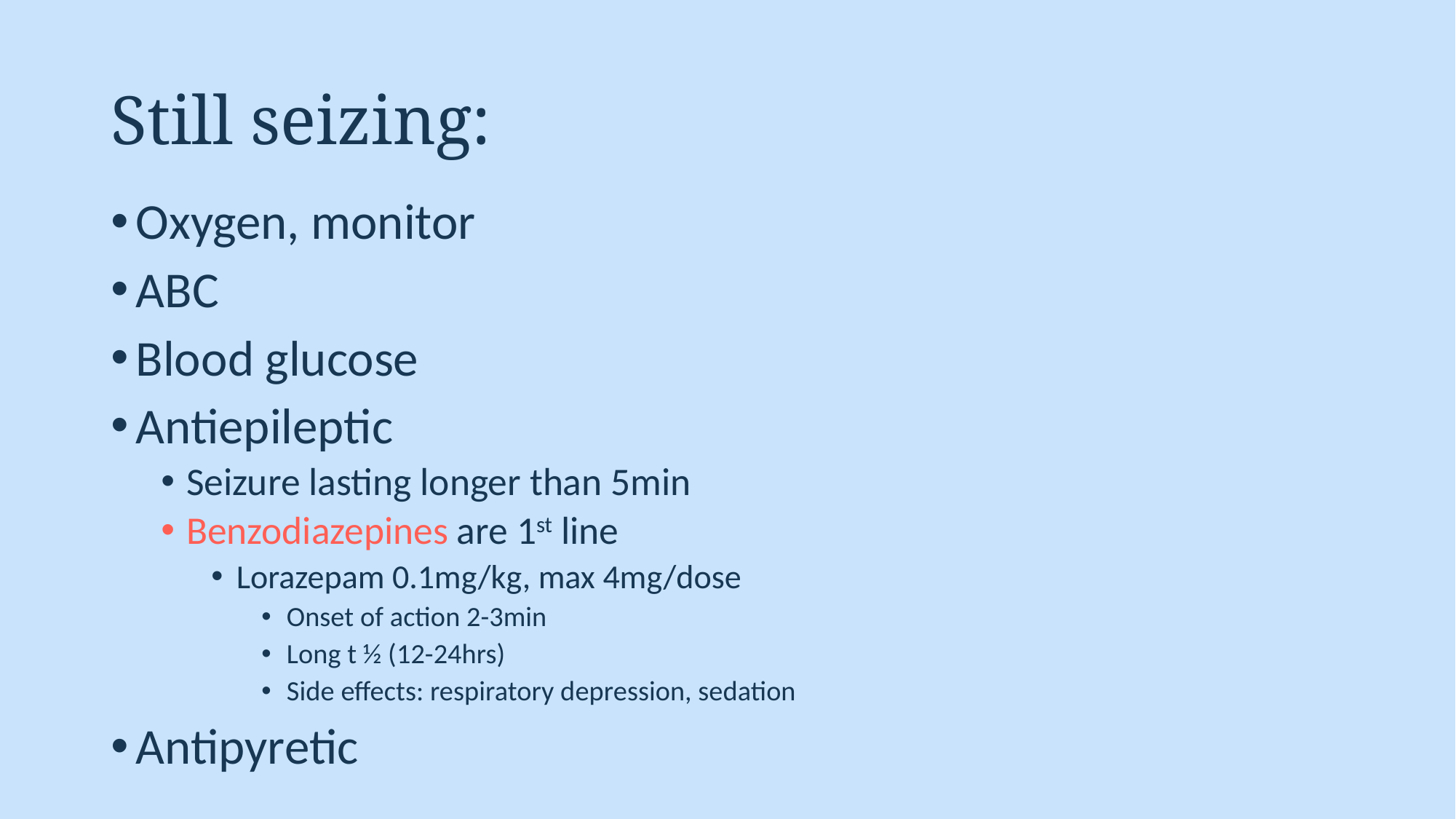

# Still seizing:
Oxygen, monitor
ABC
Blood glucose
Antiepileptic
Seizure lasting longer than 5min
Benzodiazepines are 1st line
Lorazepam 0.1mg/kg, max 4mg/dose
Onset of action 2-3min
Long t ½ (12-24hrs)
Side effects: respiratory depression, sedation
Antipyretic

## Slide 16
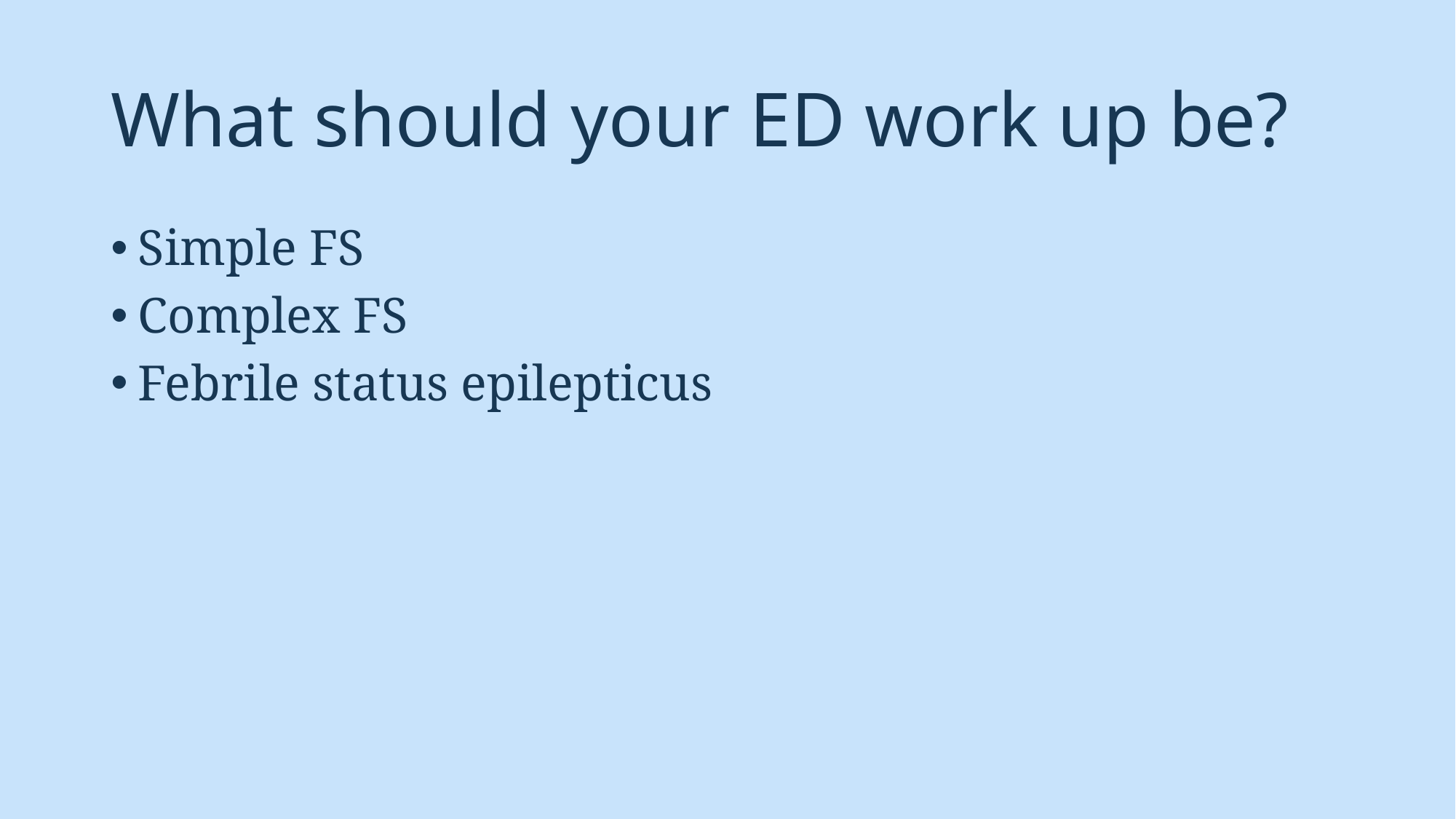

# What should your ED work up be?
Simple FS
Complex FS
Febrile status epilepticus

## Slide 17
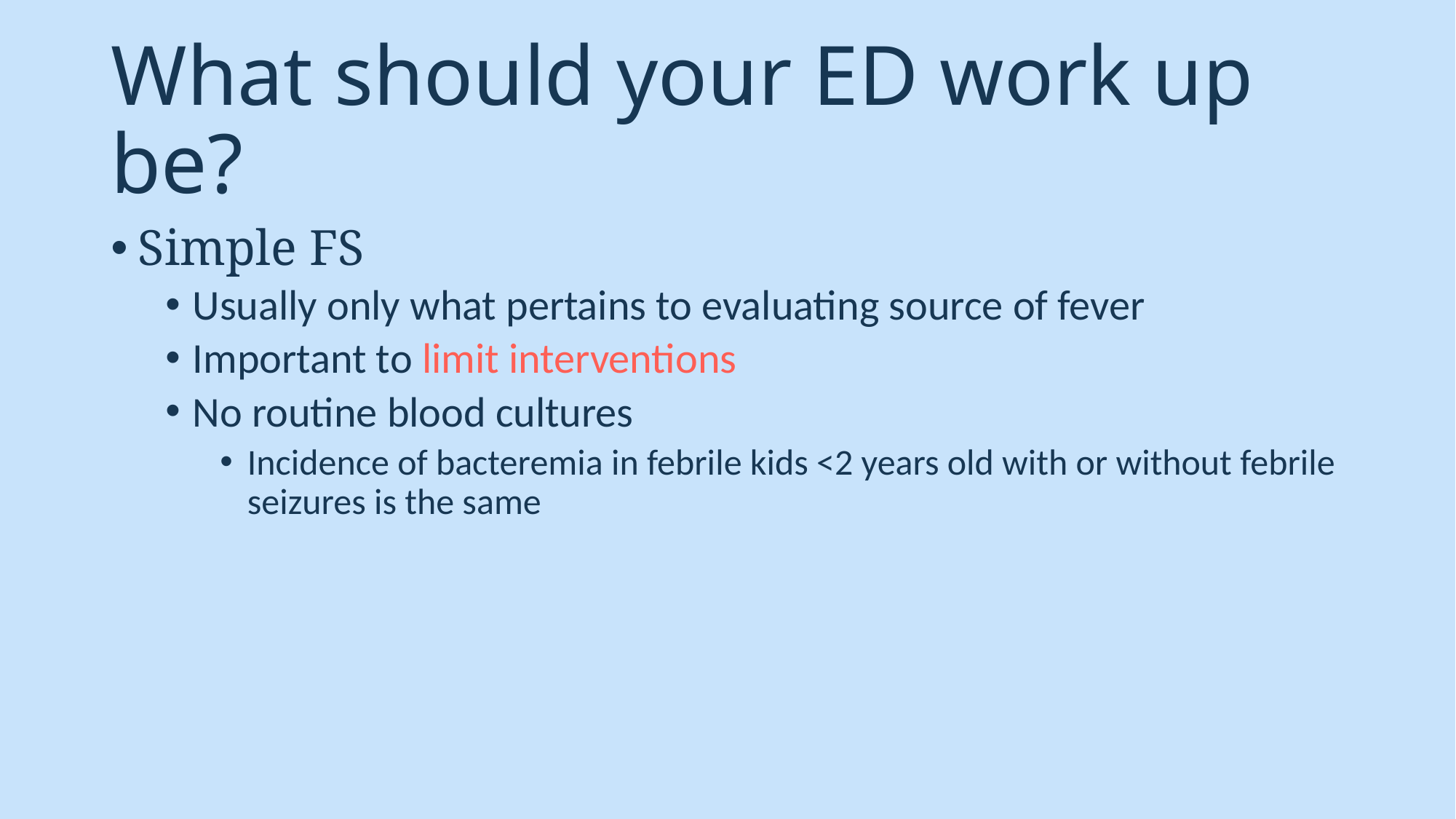

# What should your ED work up be?
Simple FS
Usually only what pertains to evaluating source of fever
Important to limit interventions
No routine blood cultures
Incidence of bacteremia in febrile kids <2 years old with or without febrile seizures is the same

## Slide 18
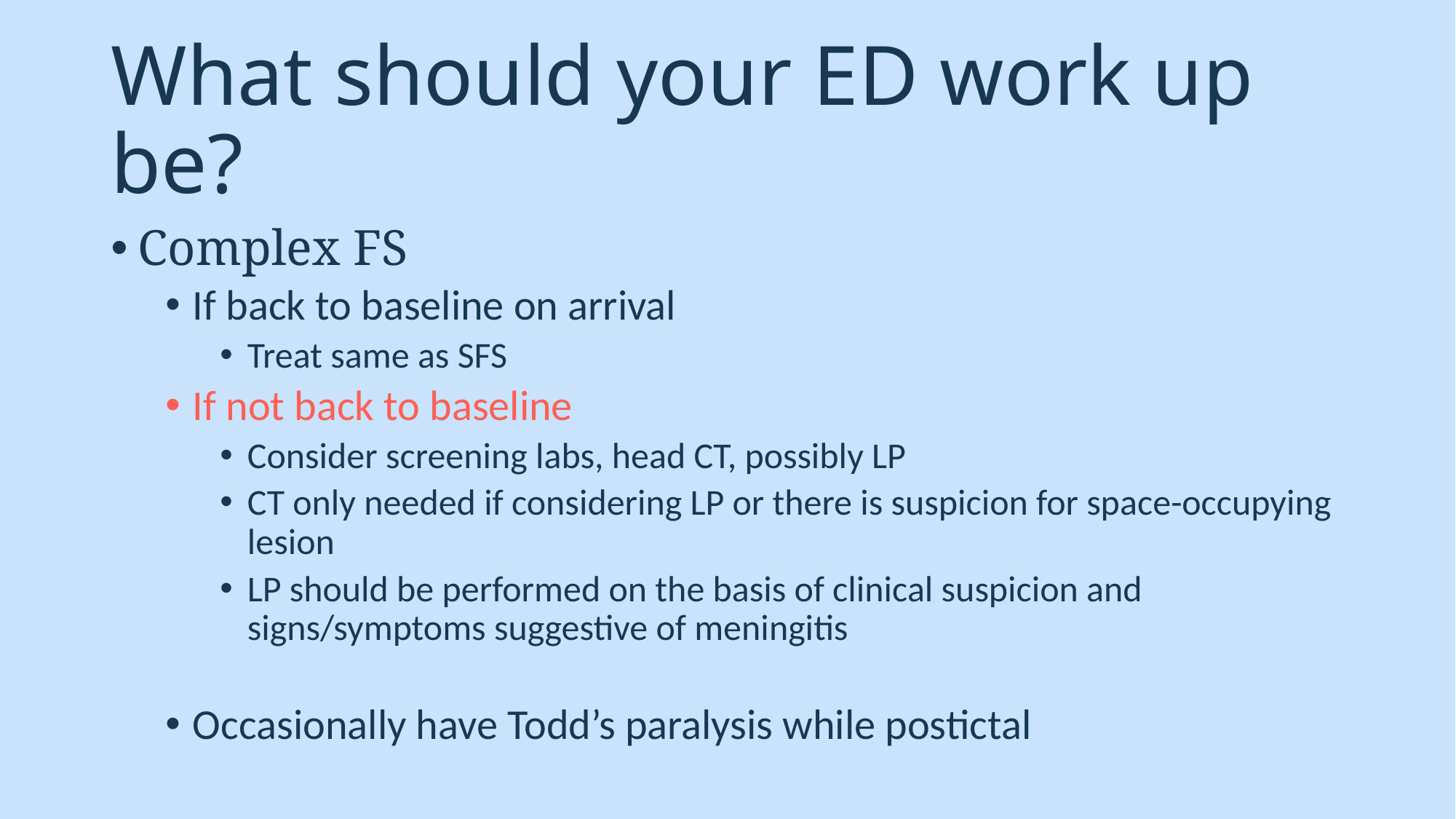

# What should your ED work up be?
Complex FS
If back to baseline on arrival
Treat same as SFS
If not back to baseline
Consider screening labs, head CT, possibly LP
CT only needed if considering LP or there is suspicion for space-occupying lesion
LP should be performed on the basis of clinical suspicion and signs/symptoms suggestive of meningitis
Occasionally have Todd’s paralysis while postictal

## Slide 19
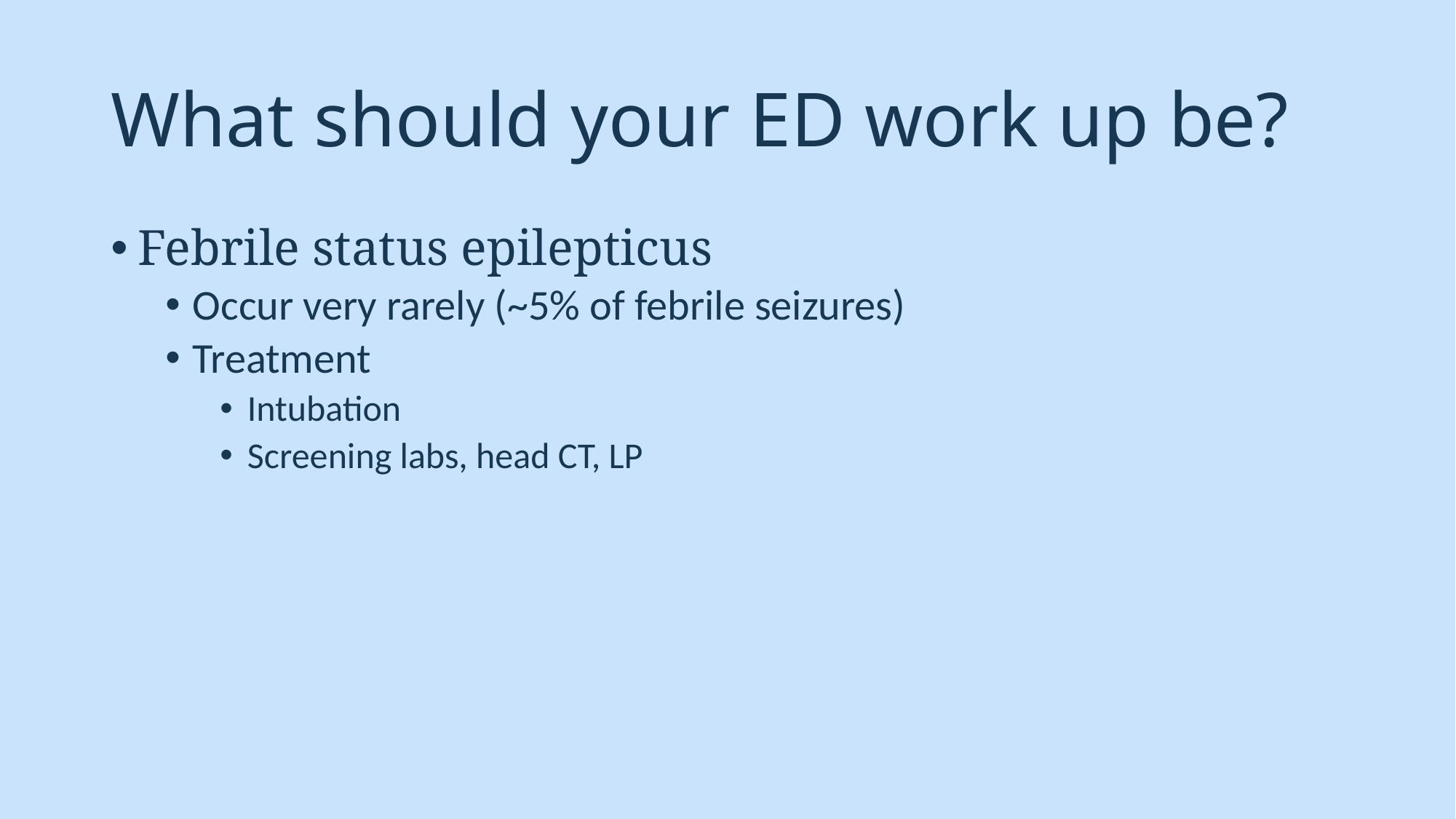

# What should your ED work up be?
Febrile status epilepticus
Occur very rarely (~5% of febrile seizures)
Treatment
Intubation
Screening labs, head CT, LP

## Slide 20
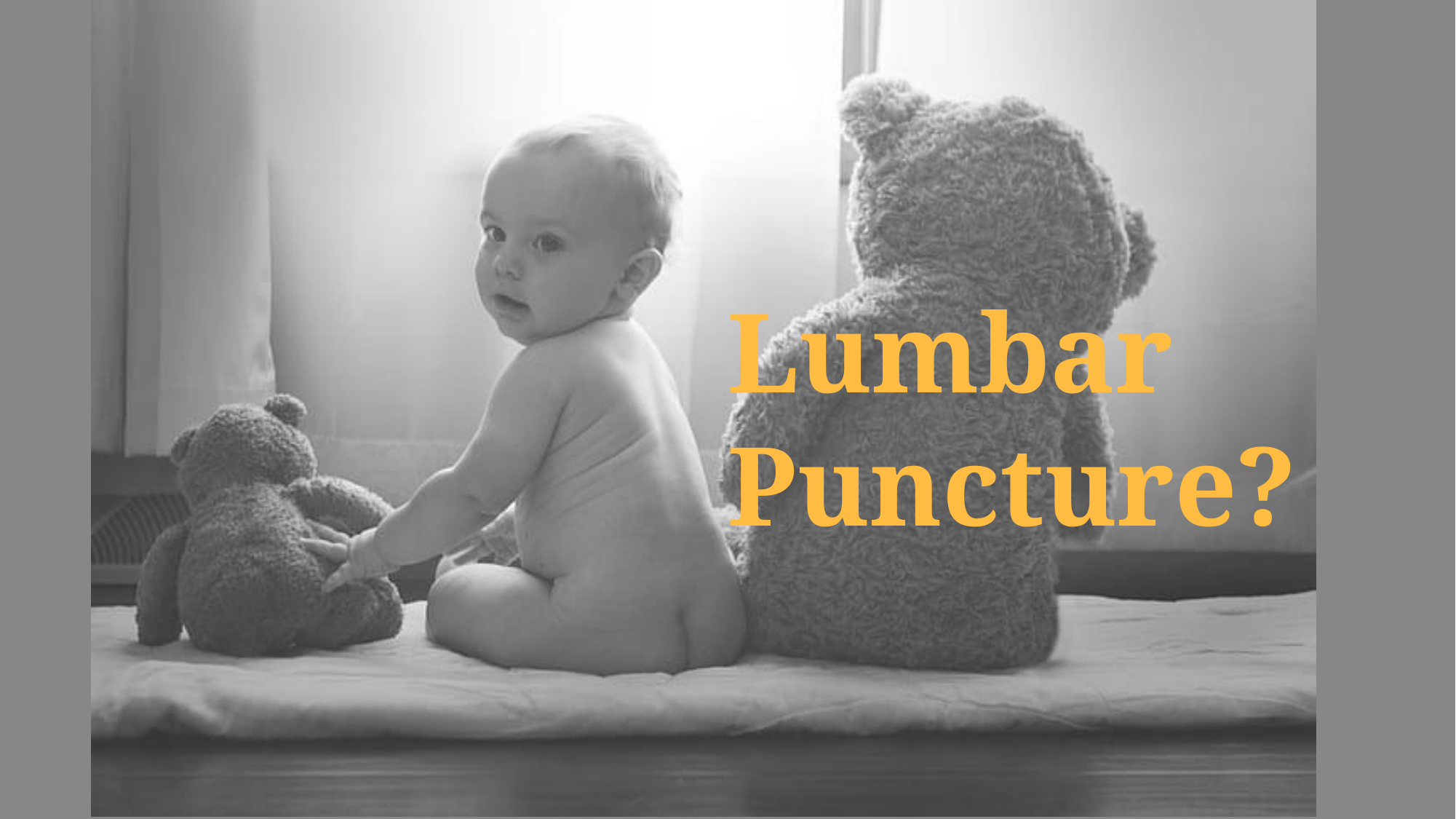

Lumbar
Puncture?

## Slide 21
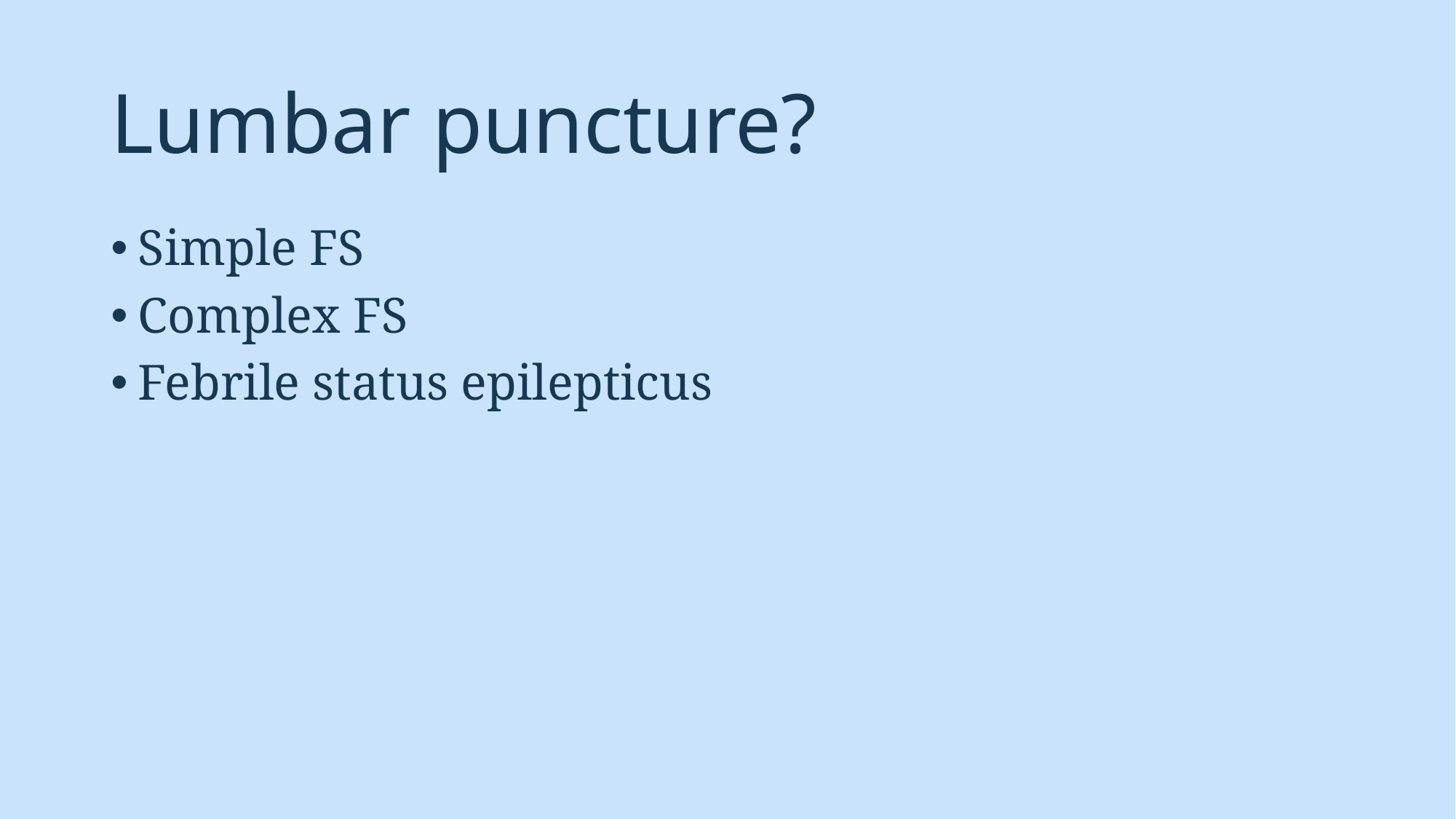

# Lumbar puncture?
Simple FS
Complex FS
Febrile status epilepticus

## Slide 22
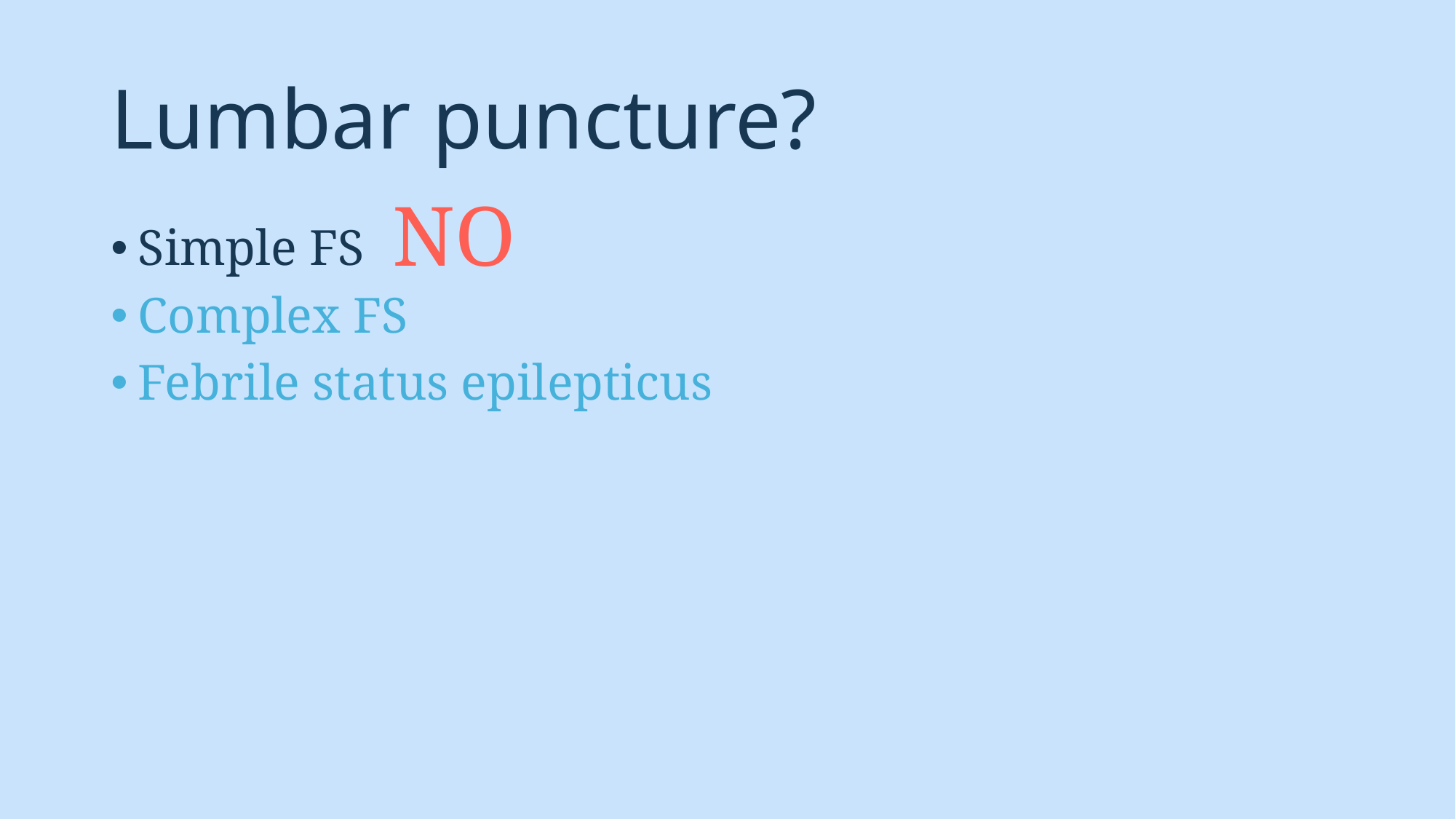

# Lumbar puncture?
NO
Simple FS
Complex FS
Febrile status epilepticus

## Slide 23
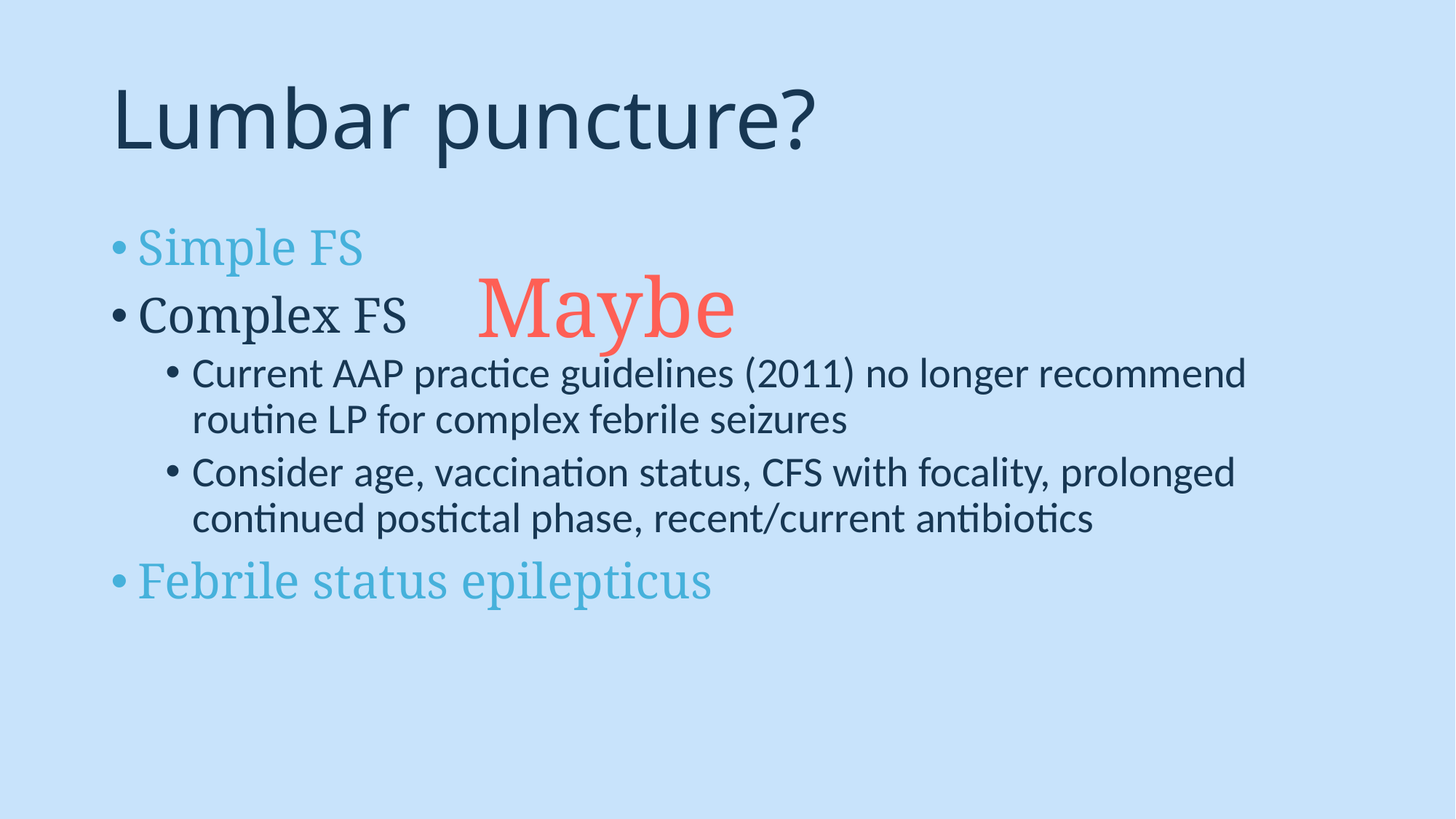

# Lumbar puncture?
Simple FS
Complex FS
Current AAP practice guidelines (2011) no longer recommend routine LP for complex febrile seizures
Consider age, vaccination status, CFS with focality, prolonged continued postictal phase, recent/current antibiotics
Febrile status epilepticus
Maybe

## Slide 24
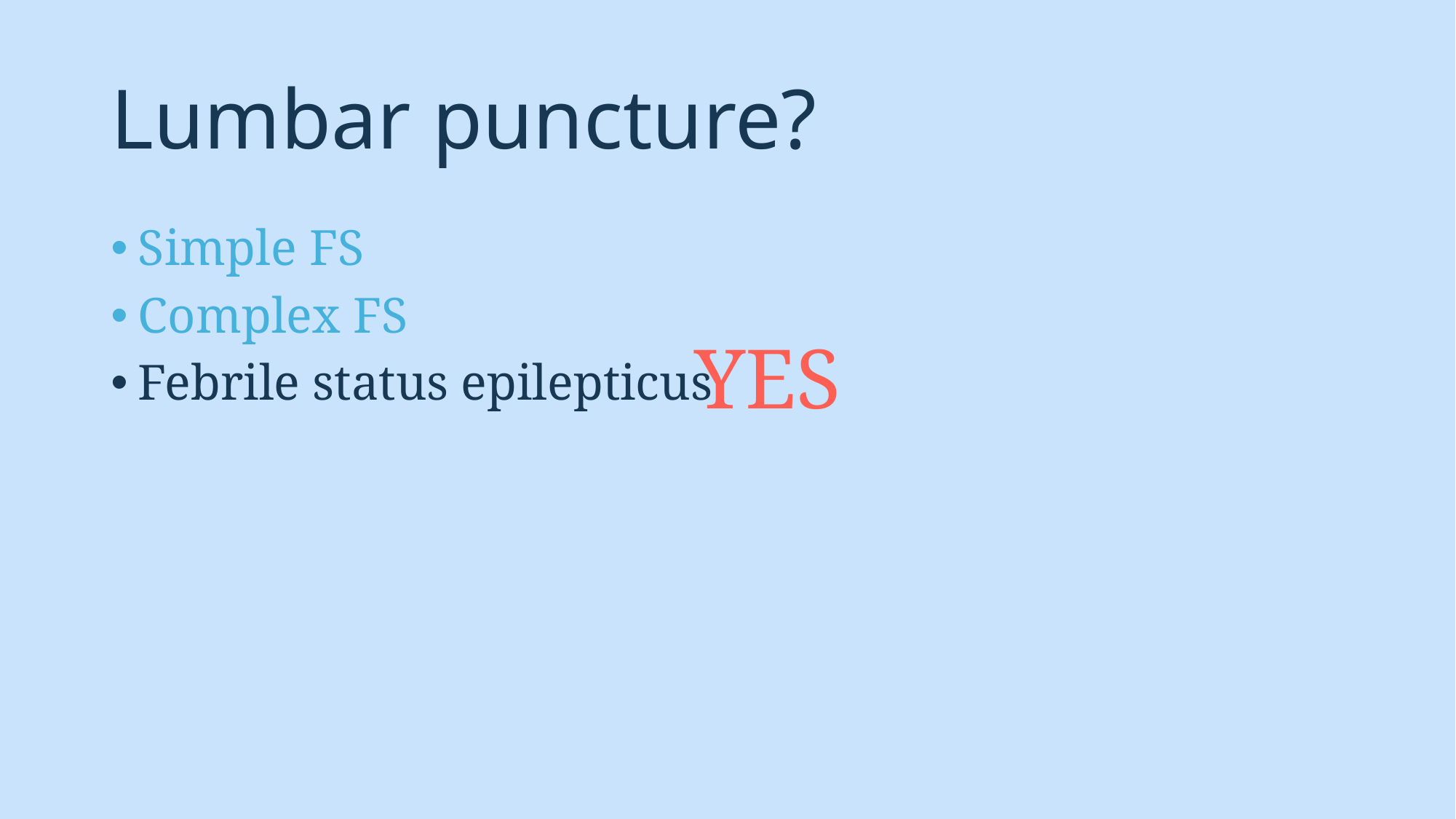

# Lumbar puncture?
Simple FS
Complex FS
Febrile status epilepticus
YES

## Slide 25
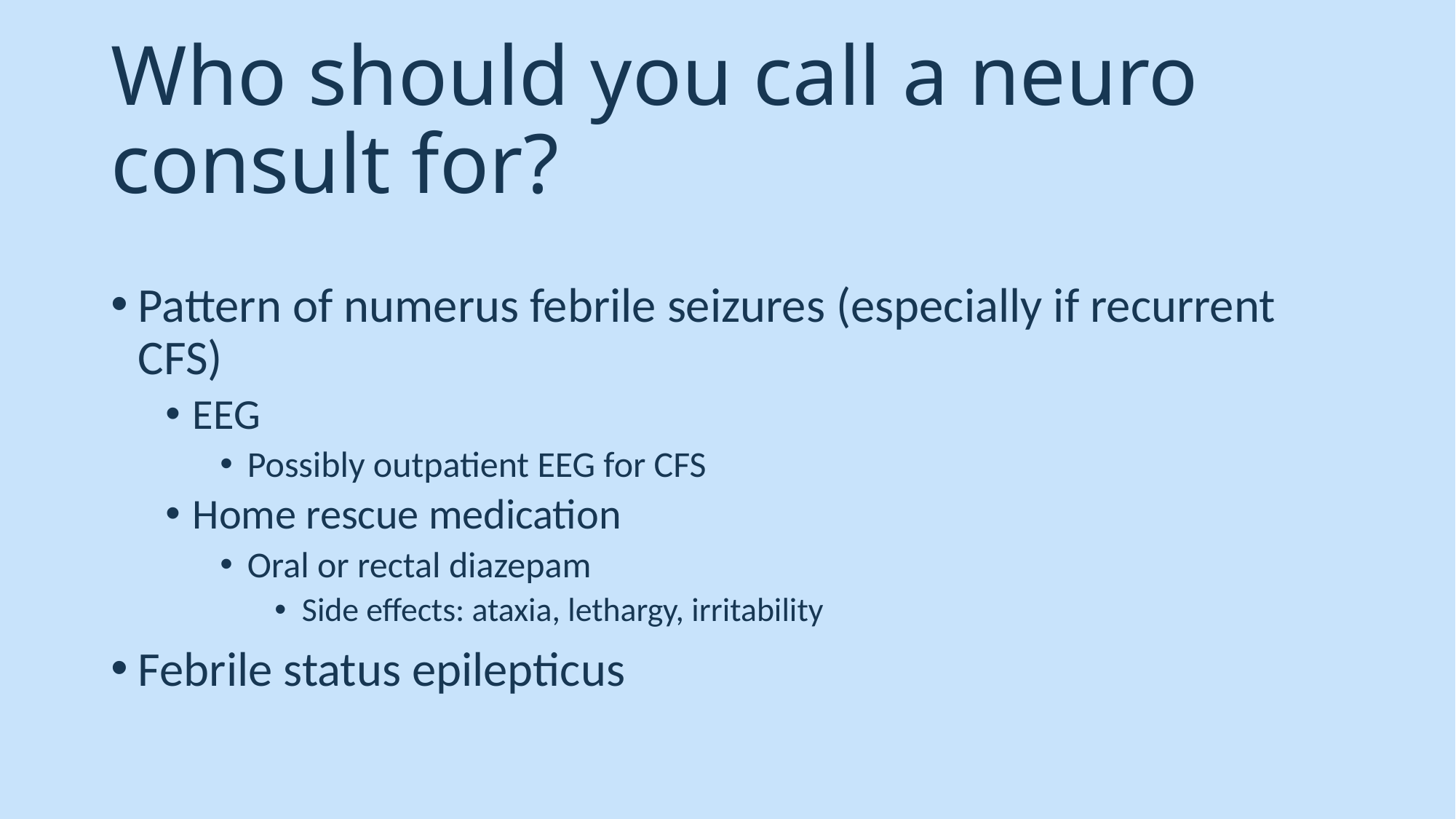

# Who should you call a neuro consult for?
Pattern of numerus febrile seizures (especially if recurrent CFS)
EEG
Possibly outpatient EEG for CFS
Home rescue medication
Oral or rectal diazepam
Side effects: ataxia, lethargy, irritability
Febrile status epilepticus

## Slide 26
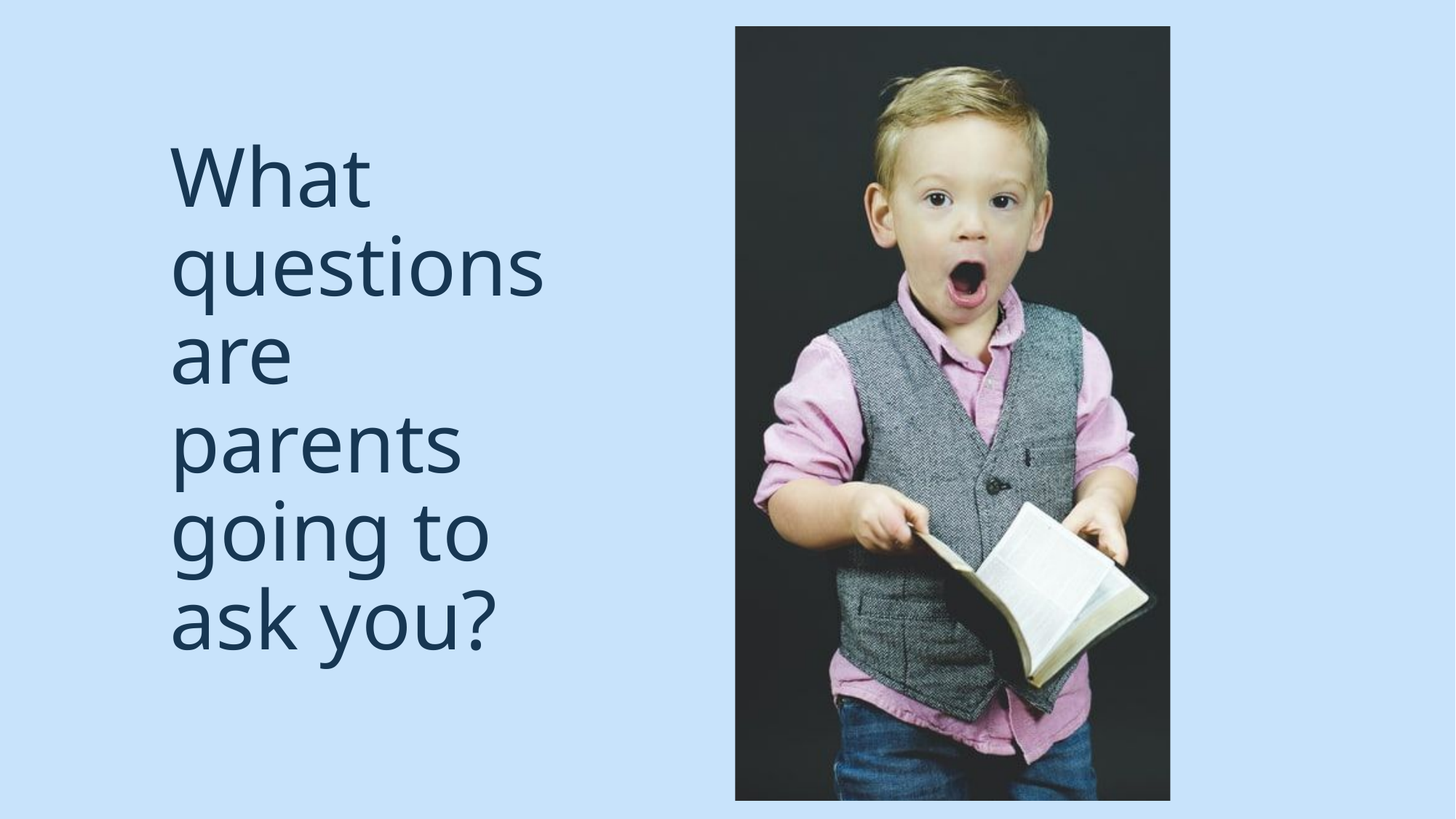

# What questions are parents going to ask you?

## Slide 27
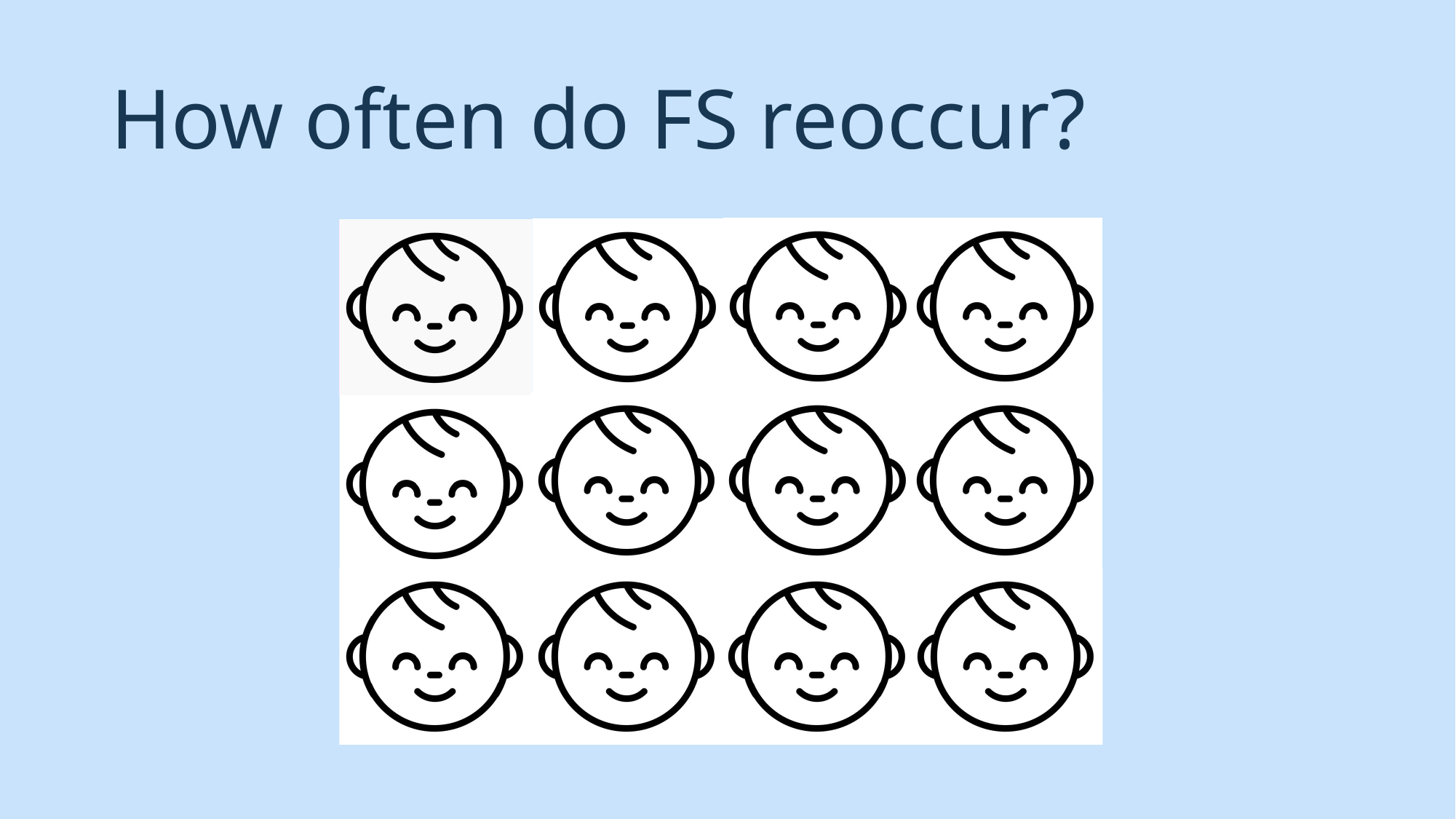

# How often do FS reoccur?

## Slide 28
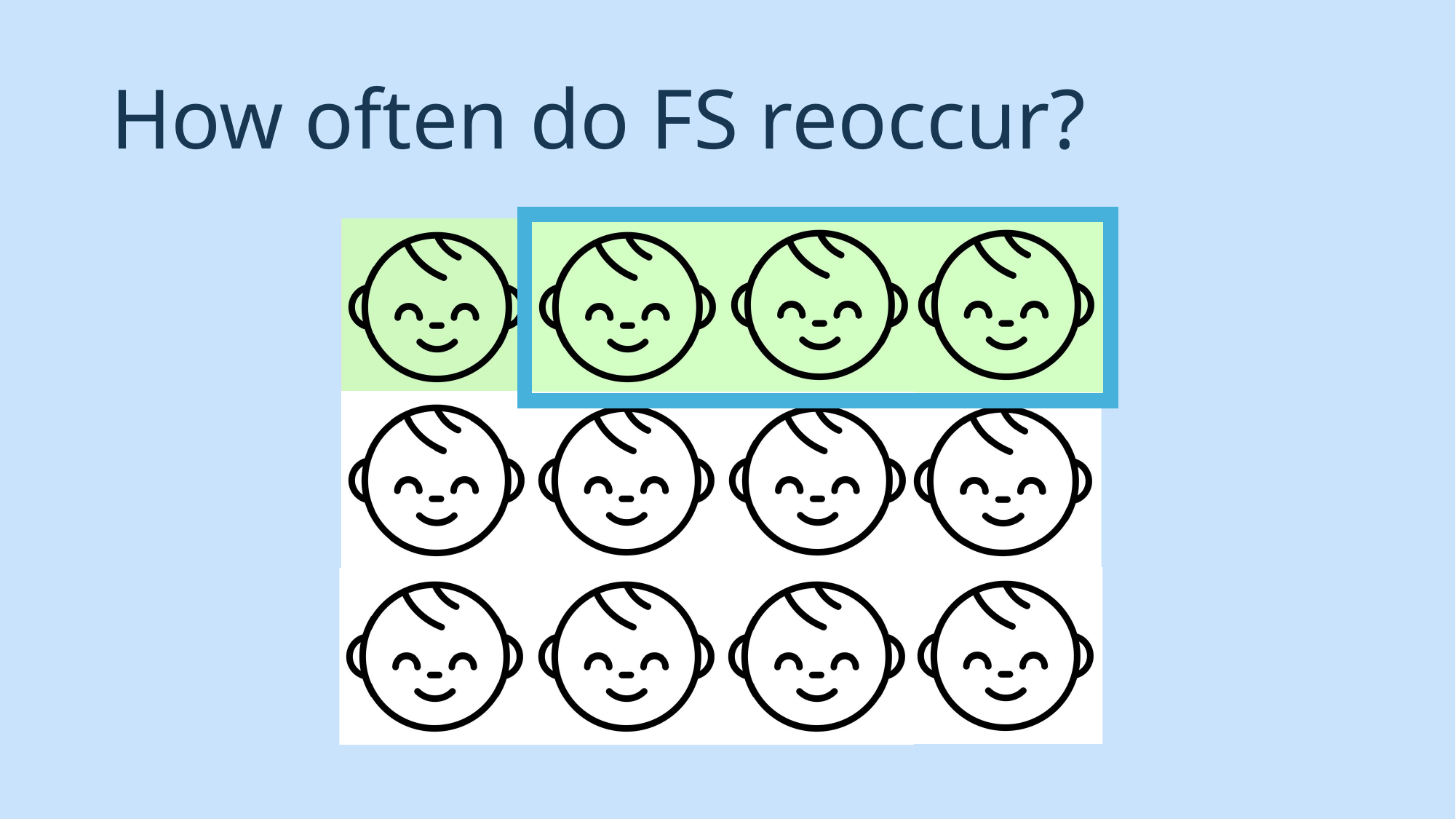

# How often do FS reoccur?

## Slide 29
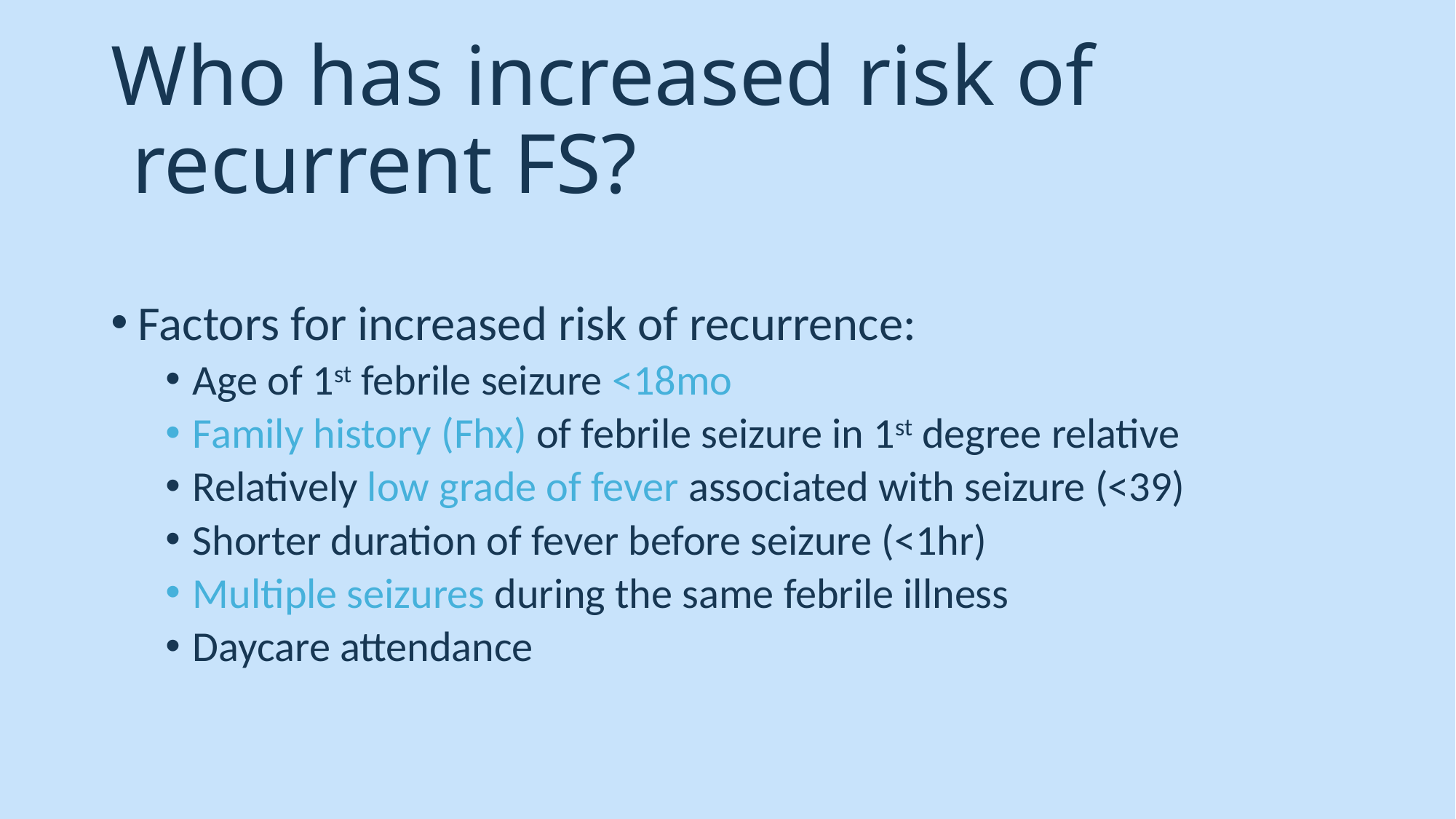

# Who has increased risk of recurrent FS?
Factors for increased risk of recurrence:
Age of 1st febrile seizure <18mo
Family history (Fhx) of febrile seizure in 1st degree relative
Relatively low grade of fever associated with seizure (<39)
Shorter duration of fever before seizure (<1hr)
Multiple seizures during the same febrile illness
Daycare attendance

## Slide 30
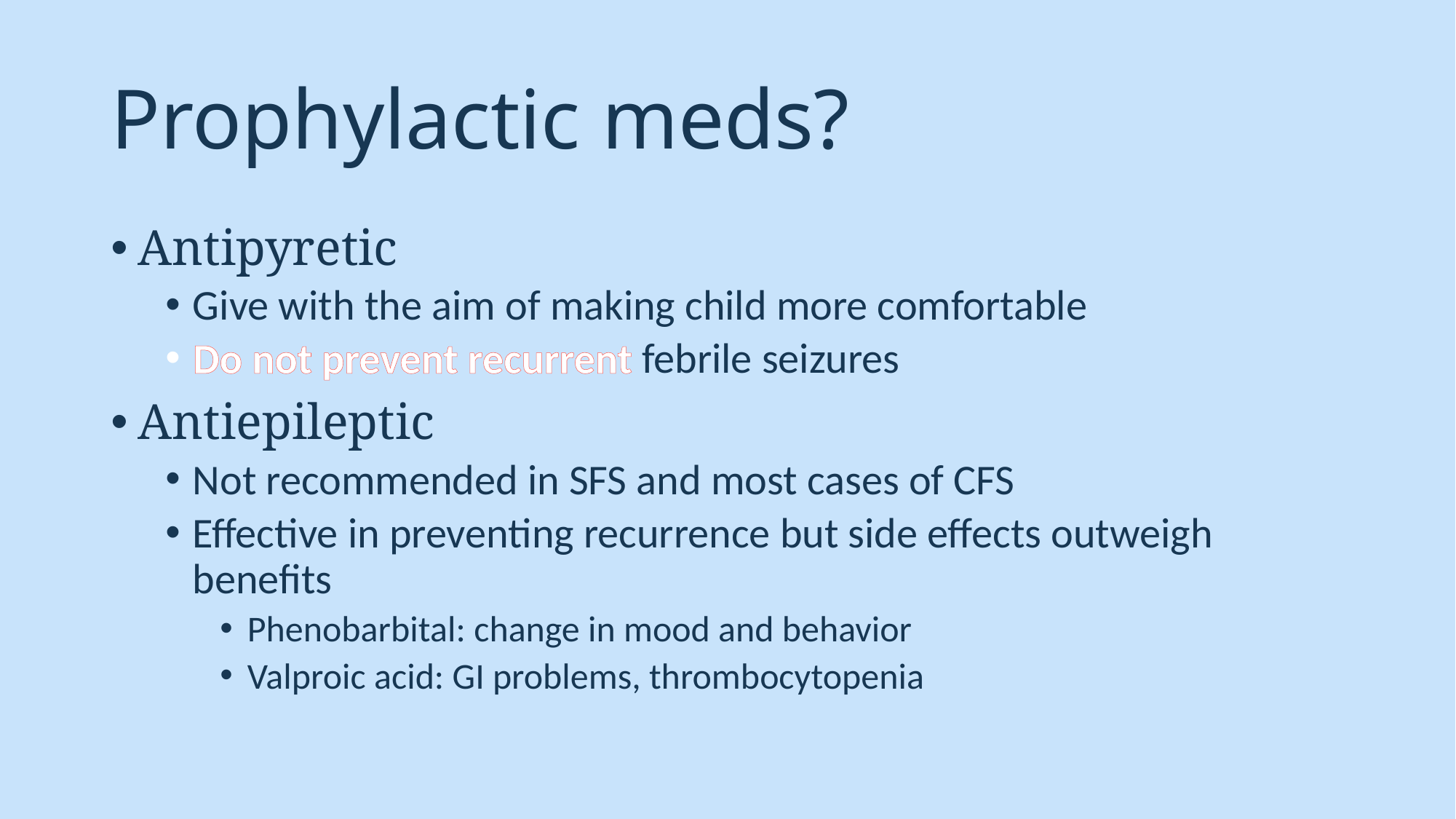

# Prophylactic meds?
Antipyretic
Give with the aim of making child more comfortable
Do not prevent recurrent febrile seizures
Antiepileptic
Not recommended in SFS and most cases of CFS
Effective in preventing recurrence but side effects outweigh benefits
Phenobarbital: change in mood and behavior
Valproic acid: GI problems, thrombocytopenia

## Slide 31
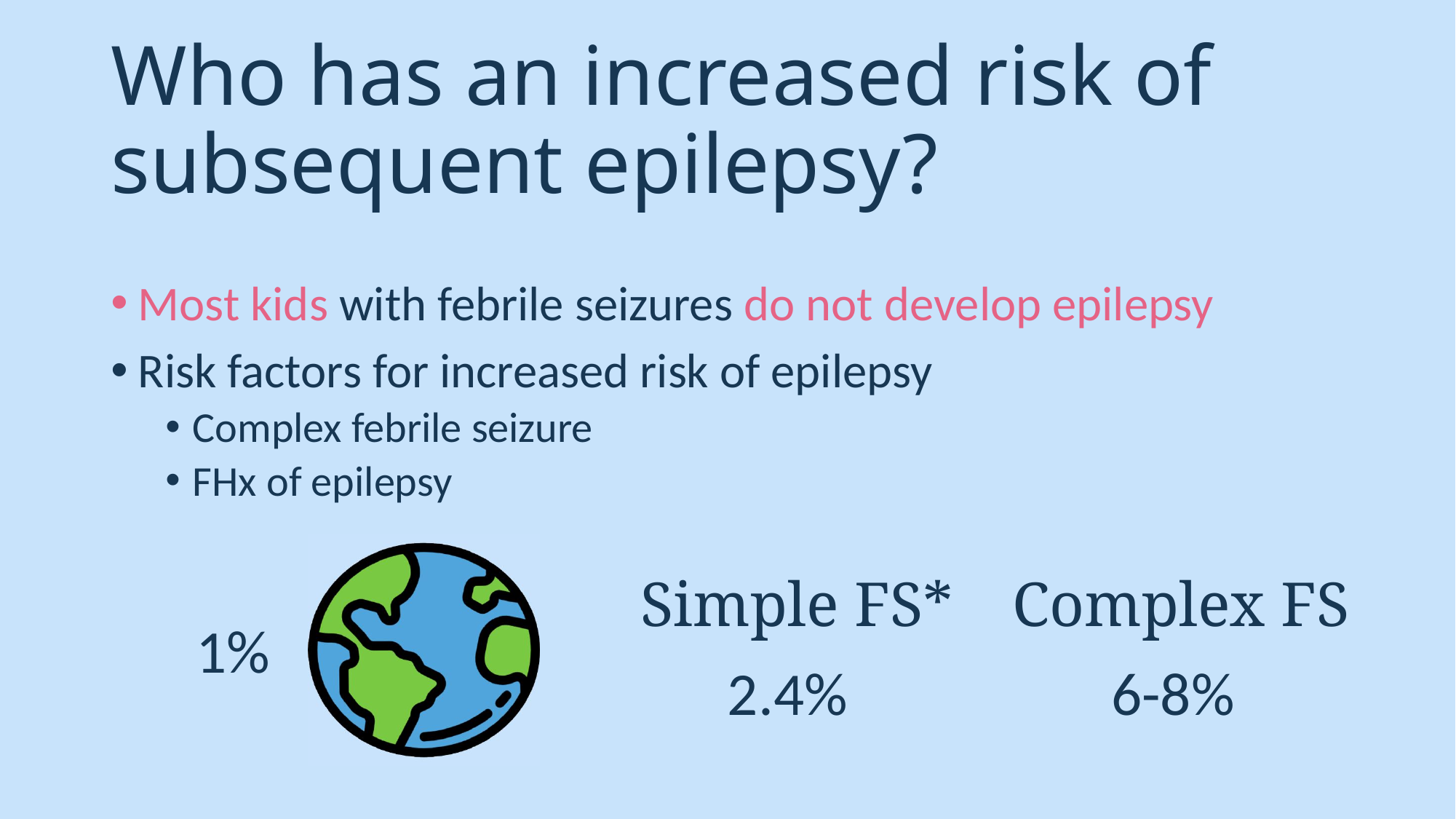

# Who has an increased risk of subsequent epilepsy?
Most kids with febrile seizures do not develop epilepsy
Risk factors for increased risk of epilepsy
Complex febrile seizure
FHx of epilepsy
Simple FS*
Complex FS
1%
2.4%
6-8%

## Slide 32
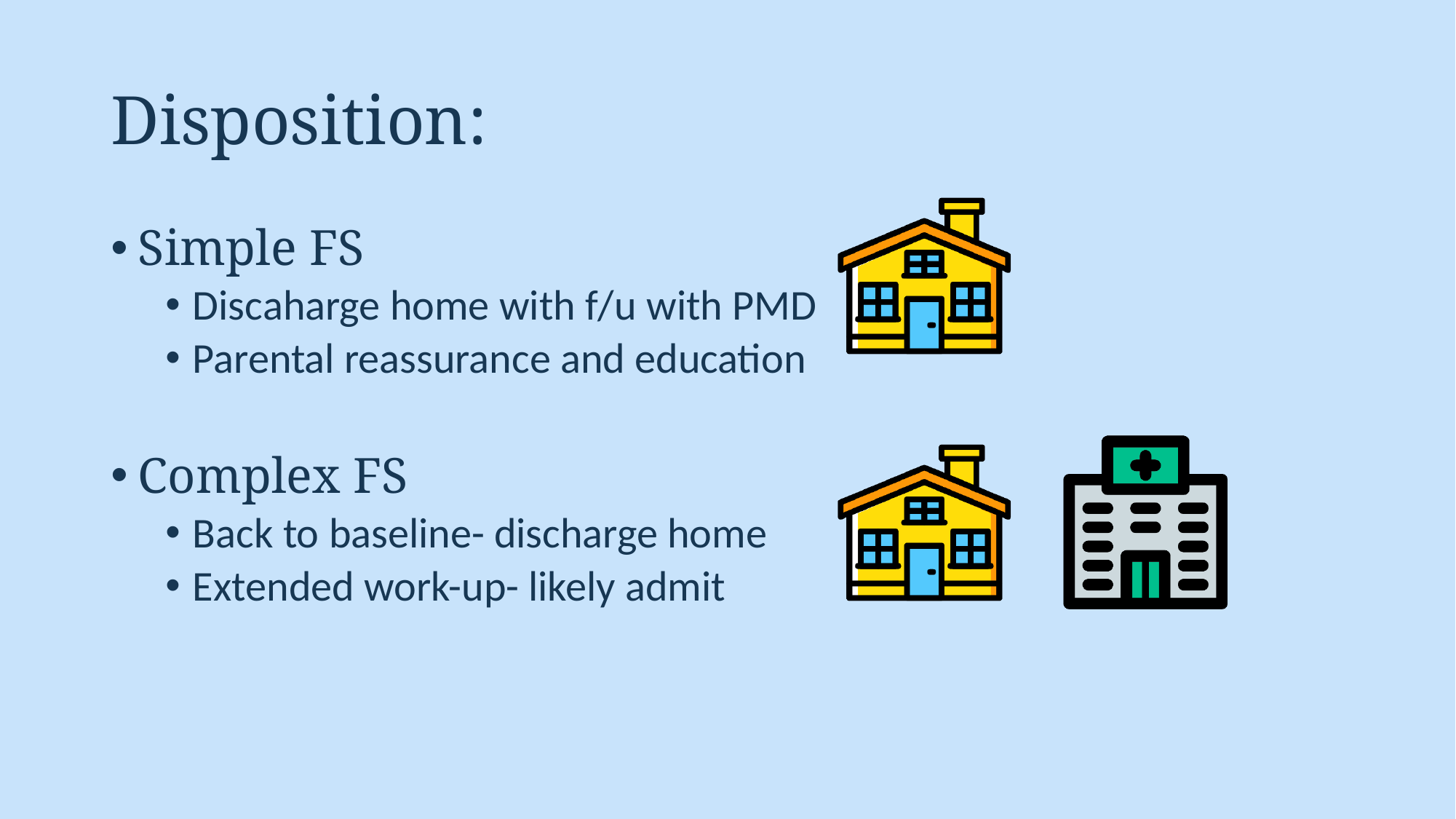

# Disposition:
Simple FS
Discaharge home with f/u with PMD
Parental reassurance and education
Complex FS
Back to baseline- discharge home
Extended work-up- likely admit

## Slide 33
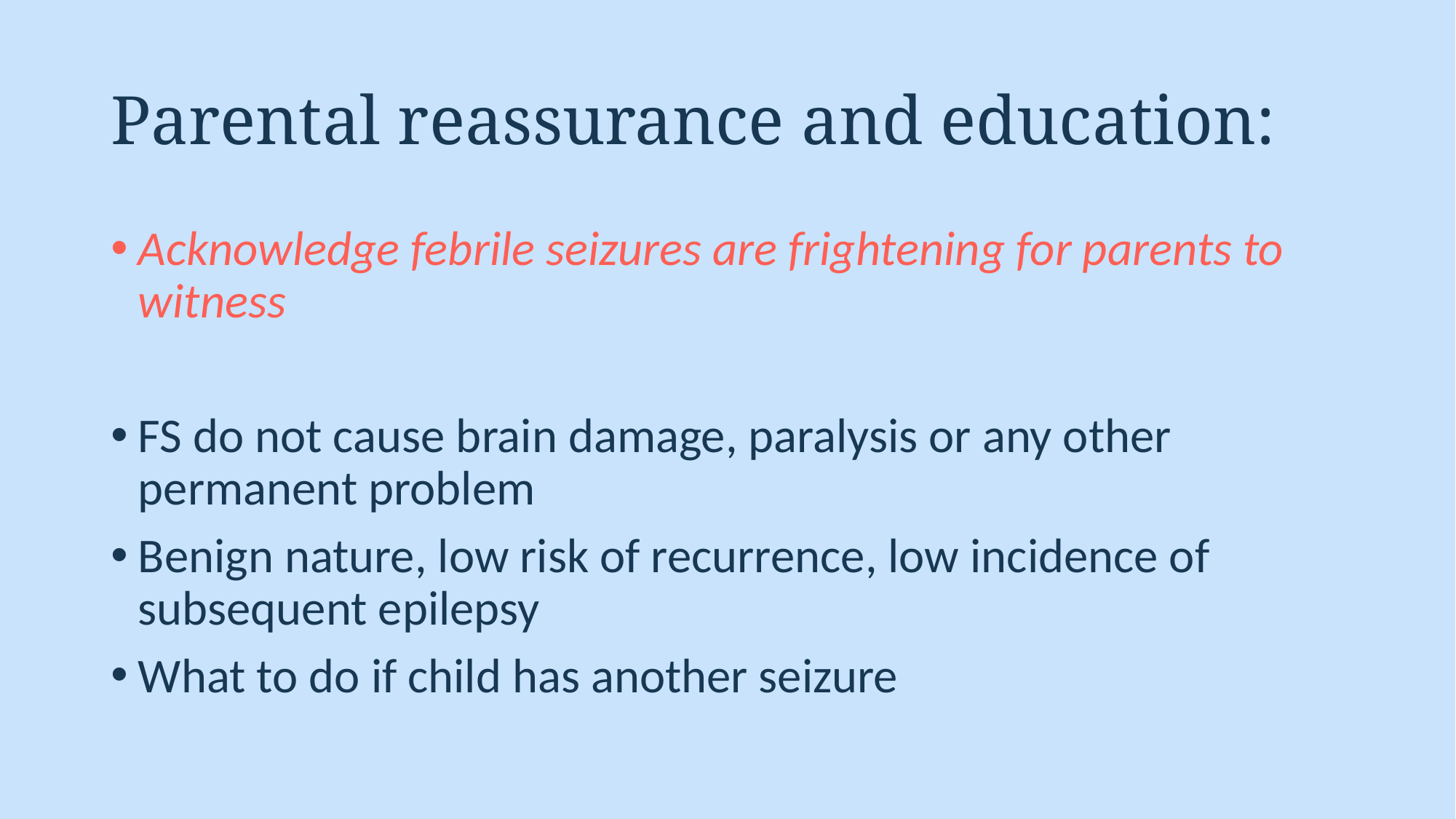

# Parental reassurance and education:
Acknowledge febrile seizures are frightening for parents to witness
FS do not cause brain damage, paralysis or any other permanent problem
Benign nature, low risk of recurrence, low incidence of subsequent epilepsy
What to do if child has another seizure

## Slide 34
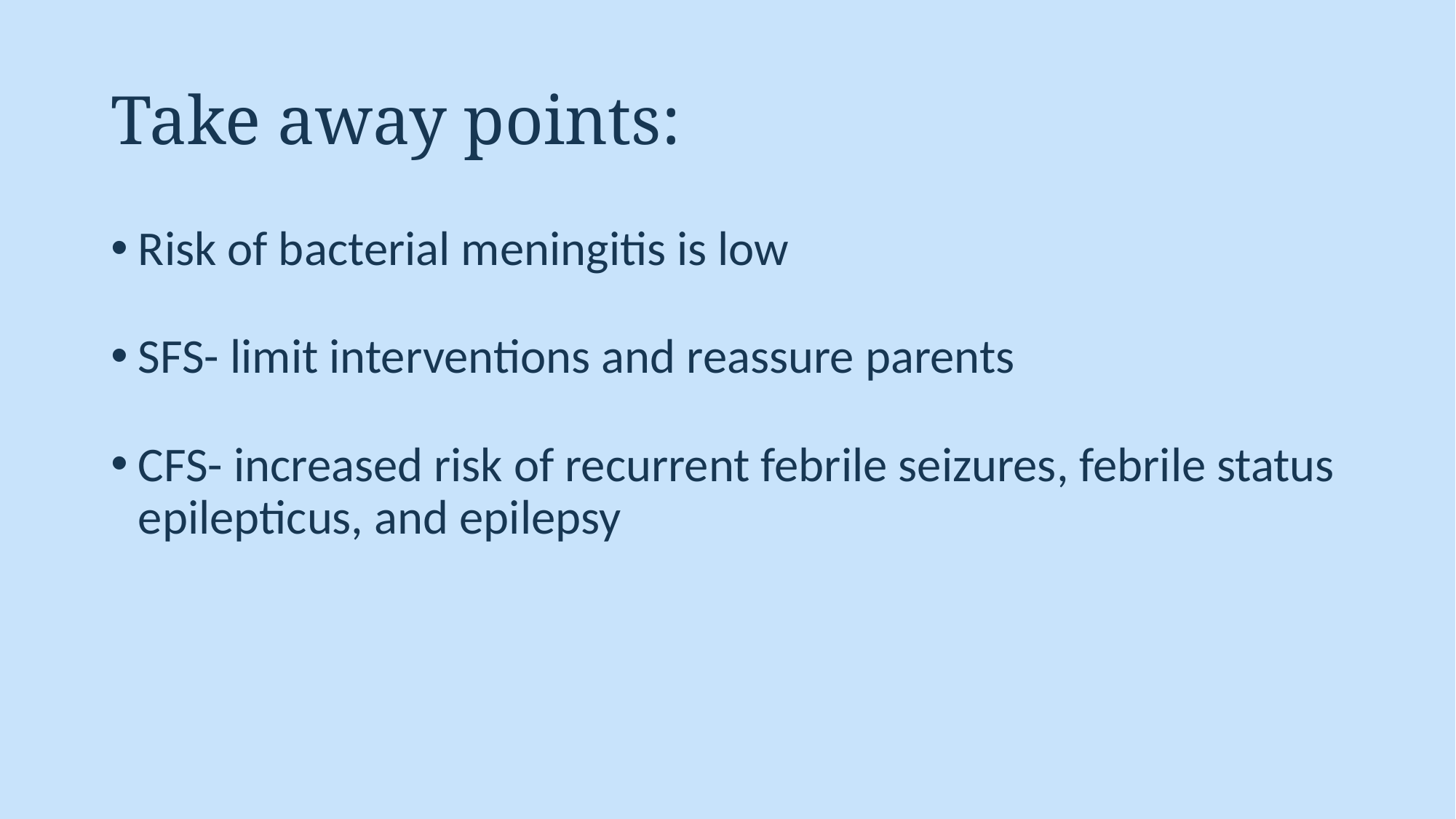

# Take away points:
Risk of bacterial meningitis is low
SFS- limit interventions and reassure parents
CFS- increased risk of recurrent febrile seizures, febrile status epilepticus, and epilepsy

## Slide 35
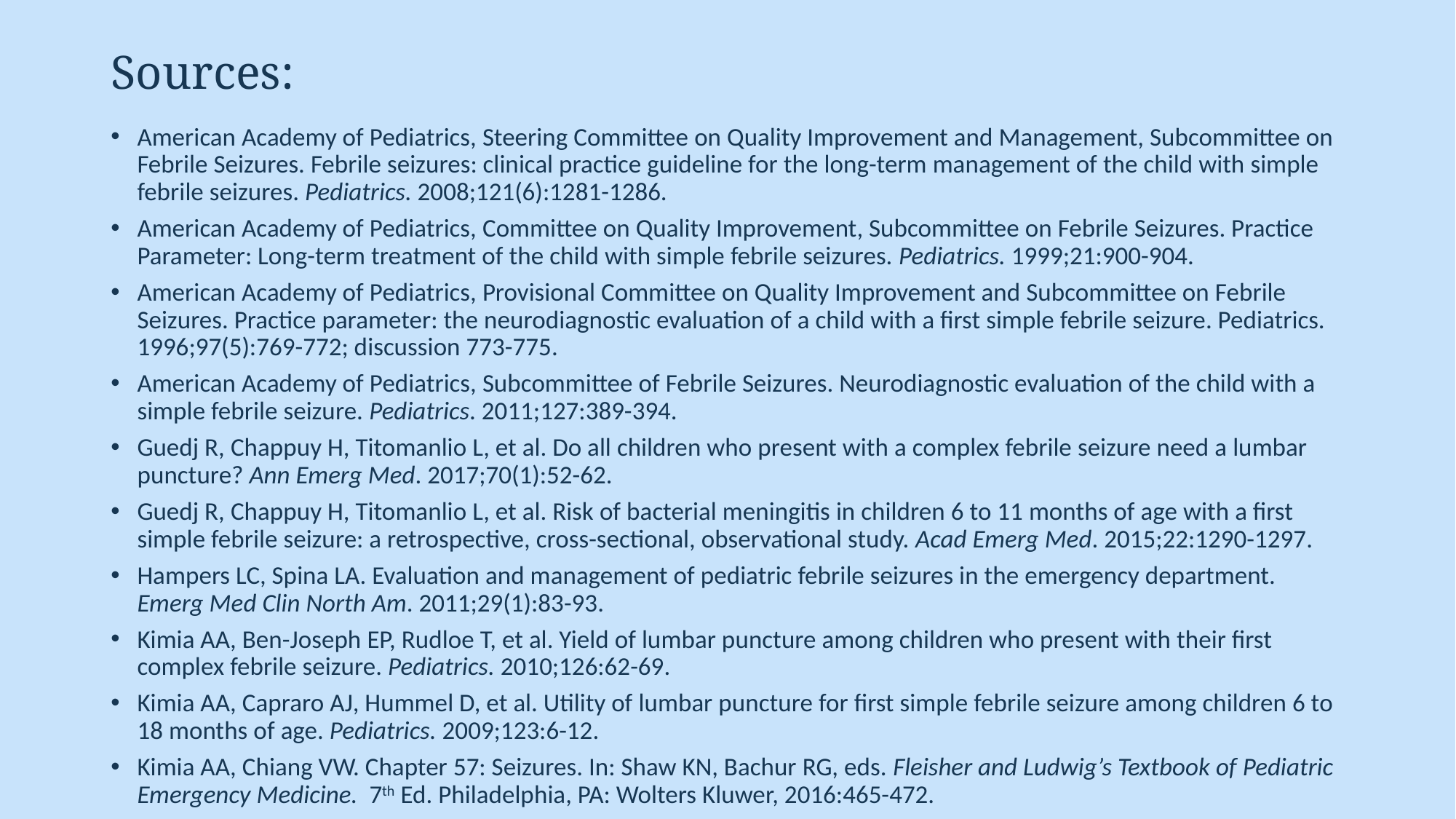

# Sources:
American Academy of Pediatrics, Steering Committee on Quality Improvement and Management, Subcommittee on Febrile Seizures. Febrile seizures: clinical practice guideline for the long-term management of the child with simple febrile seizures. Pediatrics. 2008;121(6):1281-1286.
American Academy of Pediatrics, Committee on Quality Improvement, Subcommittee on Febrile Seizures. Practice Parameter: Long-term treatment of the child with simple febrile seizures. Pediatrics. 1999;21:900-904.
American Academy of Pediatrics, Provisional Committee on Quality Improvement and Subcommittee on Febrile Seizures. Practice parameter: the neurodiagnostic evaluation of a child with a first simple febrile seizure. Pediatrics. 1996;97(5):769-772; discussion 773-775.
American Academy of Pediatrics, Subcommittee of Febrile Seizures. Neurodiagnostic evaluation of the child with a simple febrile seizure. Pediatrics. 2011;127:389-394.
Guedj R, Chappuy H, Titomanlio L, et al. Do all children who present with a complex febrile seizure need a lumbar puncture? Ann Emerg Med. 2017;70(1):52-62.
Guedj R, Chappuy H, Titomanlio L, et al. Risk of bacterial meningitis in children 6 to 11 months of age with a first simple febrile seizure: a retrospective, cross-sectional, observational study. Acad Emerg Med. 2015;22:1290-1297.
Hampers LC, Spina LA. Evaluation and management of pediatric febrile seizures in the emergency department. Emerg Med Clin North Am. 2011;29(1):83-93.
Kimia AA, Ben-Joseph EP, Rudloe T, et al. Yield of lumbar puncture among children who present with their first complex febrile seizure. Pediatrics. 2010;126:62-69.
Kimia AA, Capraro AJ, Hummel D, et al. Utility of lumbar puncture for first simple febrile seizure among children 6 to 18 months of age. Pediatrics. 2009;123:6-12.
Kimia AA, Chiang VW. Chapter 57: Seizures. In: Shaw KN, Bachur RG, eds. Fleisher and Ludwig’s Textbook of Pediatric Emergency Medicine. 7th Ed. Philadelphia, PA: Wolters Kluwer, 2016:465-472.

## Slide 36
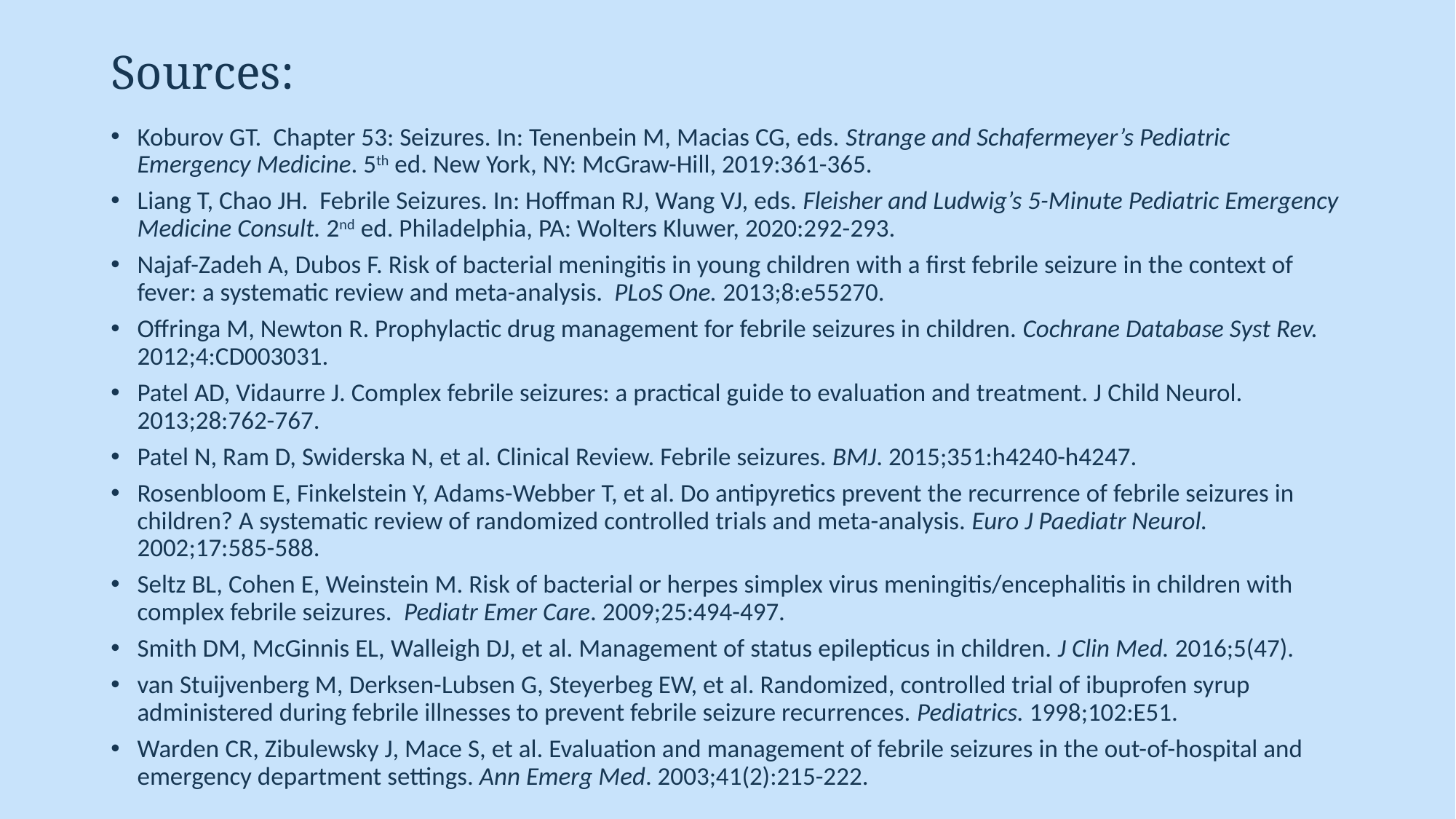

# Sources:
Koburov GT. Chapter 53: Seizures. In: Tenenbein M, Macias CG, eds. Strange and Schafermeyer’s Pediatric Emergency Medicine. 5th ed. New York, NY: McGraw-Hill, 2019:361-365.
Liang T, Chao JH. Febrile Seizures. In: Hoffman RJ, Wang VJ, eds. Fleisher and Ludwig’s 5-Minute Pediatric Emergency Medicine Consult. 2nd ed. Philadelphia, PA: Wolters Kluwer, 2020:292-293.
Najaf-Zadeh A, Dubos F. Risk of bacterial meningitis in young children with a first febrile seizure in the context of fever: a systematic review and meta-analysis. PLoS One. 2013;8:e55270.
Offringa M, Newton R. Prophylactic drug management for febrile seizures in children. Cochrane Database Syst Rev. 2012;4:CD003031.
Patel AD, Vidaurre J. Complex febrile seizures: a practical guide to evaluation and treatment. J Child Neurol. 2013;28:762-767.
Patel N, Ram D, Swiderska N, et al. Clinical Review. Febrile seizures. BMJ. 2015;351:h4240-h4247.
Rosenbloom E, Finkelstein Y, Adams-Webber T, et al. Do antipyretics prevent the recurrence of febrile seizures in children? A systematic review of randomized controlled trials and meta-analysis. Euro J Paediatr Neurol. 2002;17:585-588.
Seltz BL, Cohen E, Weinstein M. Risk of bacterial or herpes simplex virus meningitis/encephalitis in children with complex febrile seizures. Pediatr Emer Care. 2009;25:494-497.
Smith DM, McGinnis EL, Walleigh DJ, et al. Management of status epilepticus in children. J Clin Med. 2016;5(47).
van Stuijvenberg M, Derksen-Lubsen G, Steyerbeg EW, et al. Randomized, controlled trial of ibuprofen syrup administered during febrile illnesses to prevent febrile seizure recurrences. Pediatrics. 1998;102:E51.
Warden CR, Zibulewsky J, Mace S, et al. Evaluation and management of febrile seizures in the out-of-hospital and emergency department settings. Ann Emerg Med. 2003;41(2):215-222.
